# Reproducibility and quality of hypertrophy-related training plans generated by GPT-4 and Google Gemini as evaluated by coaching experts

**DOI:** 10.5114/biolsport.2025.145911

**Published:** 2024-12-18

**Authors:** Tim Havers, Lukas Masur, Eduard Isenmann, Stephan Geisler, Christoph Zinner, Billy Sperlich, Peter Düking

**Affiliations:** 1 Department of Fitness and Health, IST University of Applied Sciences, Düsseldorf, Germany; 2 Faculty of Sport and Health Sciences, Technical University of Munich, Munich, Germany; 3 Department of Sports Science and Movement Pedagogy, Technische Universität Braunschweig, Braunschweig, Germany; 4 Department of Molecular and Cellular Sports Medicine, Institute for Cardiovascular Research and Sports Medicine, German Sport University Cologne, Cologne; 5 Department of Sport, University of Applied Sciences for Police and Administration of Hesse, Wiesbaden, Germany; 6 Integrative and Experimental Exercise Science and Training, Institute of Sport Science, University of Würzburg, Germany

**Keywords:** Artificial intelligence, Chatbots, Digital health, Digital training, Innovation, mHealth, Technology

## Abstract

Large Language Models (LLMs) are increasingly utilized in various domains, including the generation of training plans. However, the reproducibility and quality of training plans produced by different LLMs have not been studied extensively. This study aims to: i) investigate and compare the quality of muscle hypertrophy-related resistance training (RT) plans generated by Google Gemini (GG) and GPT-4, and ii) the reproducibility of the RT plans when the same prompts are provided multiple times concomitantly. Two distinct prompts were used, one providing little information about the training plan requirements and the other providing detailed information. These prompts were input into GG and GPT-4 by two different individuals, resulting in the generation of eight RT plans. These plans were evaluated by 12 coaching experts using a 5-point Likert scale, based on quality criteria derived from the literature. The results indicated a high degree of reproducibility, as indicated by coaching expert evaluation, when the same distinct prompts were provided multiple times to the LLMs of interest, with 27 out of 28 items showing no differences (p > 0.05). Overall, GPT-4 was rated higher on several aspects of RT quality criteria (p = 0.000–0.043). Additionally, compared to little information, higher information density within the prompts resulted in higher rated RT quality (p = 0.000–0.037). Our findings show that RT plans can be generated reproducibly with the same quality when using the same prompts. Furthermore, quality improves with more detailed input, and GPT-4 outperformed GG in generating higherquality plans. These results suggest that detailed information input is crucial for LLM performance.

## INTRODUCTION

Resistance training is a key element in many sports and for physical fitness, promoting muscle hypertrophy and strength development [1]. Designing resistance training programs is a nuanced process, requiring expertise e.g. in exercise physiology [2, 3], biomechanics [4, 5] and training science [6, 7, 8]. Athletes lacking this knowledge are prone to designing erroneous strength training plans, which can result in underperformance or even health issues. This underscores the need for guidance in creating individualized strength training programs. The emergence of artificial intelligence (AI), and more specifically of Large Language Models (LLMs), has the potential to assist inexperienced athletes by providing them with well-designed strength training plans. LLMs such as GPT-4 and Google Gemini have been trained on an extensive corpus of text and enable humanlike conversational interactions in various applications by providing responses to user input [9, 10]. LLMs hold promise but also limitations in providing assistance across various disciplines, including medicine [11, 12, 13], providing health promotion [14, 15], designing endurance training plans [16], or designing resistance training plans [17]. However, their limitations and imperfections are also evident.

While AI shows potential in medicine, such as in administrative tasks and decision aids, significant limitations exist in accuracy, coherence, and transparency, raising ethical concerns [10, 11]. For example, ChatGPT, used as a psychiatric provider for imaginary patients, delivered appropriate advice for simple cases but deteriorated in quality with complex scenarios, potentially leading to dangerous outcomes [12]. In nutrition, ChatGPT can offer general dietary advice but often fails to account for specific health conditions and may not adhere to evidence-based guidelines. Additionally, in sports science, ChatGPT correctly calculated only 1 out of 4 sample sizes, with inconsistent results upon repeated prompts [13]. These limitations pose risks, particularly in health-related fields, where inaccuracies can lead to harmful outcomes.

In a sports context, experts rated ChatGPT-generated running plans using 22 criteria as suboptimal, though running plans improved with more detailed input. Similarly, Washif et al. assessed GPT3.5 and GPT-4.0’s 12-week strength training programs for intermediate and advanced lifters [17]. Despite aiming for strength development, the AI-generated plans included “high volume” hypertrophy blocks that did not align with the primary goal [17]. While strength and hypertrophy training variables overlap, optimizing muscle hypertrophy may require distinct approaches when pure strength development is the focus [8, 18].

While such research has improved our understanding of LLMs capability of providing recommendations for training plans, it is currently unknown if recommendations of contemporary and publicly available LLMs are in-line with recent scientific evidence as rated by coaching experts. To address this research gap, our study primarily aimed to investigate and compare muscle hypertrophy-focused resistance training plans generated by Google Gemini and GPT-4, as assessed by coaching experts based on evidence-based criteria. Our secondary goal was to determine whether generated training plans are reproducible if the same prompts were used multiple times concomitantly.

## MATERIALS AND METHODS

### General Design

To evaluate the hypertrophy-related resistance training programs generated by GPT-4 and Google Gemini, we based our analytical approach on existing literature from the fields of exercise and medical science [16, 19, 20, 21] adapting it to the goal and settings of our research. Specifically, we i) defined criteria of relevance for hypertrophy-related training plans, ii) established input information for publicly available LLMs, iii) generated hypertrophy-related training plans using the defined input information, and iv) involved coaching experts in the field of hypertrophy to evaluate the generated training plans based on the previously defined criteria. We specifically aimed to compare the training quality in three areas: 1) between GPT-4 and Google Gemini, 2) with little versus detailed prompt input within each LLM, and 3) with the same prompt (both little and detailed input) repeated within the same LLM.

### Definition of criteria of relevance for hypertrophy-related training plans

So far, there is no generally agreed-upon consensus on quality criteria for hypertrophy-related parameters. Thus, we defined criteria of relevance for our specific case after consulting with experts in resistance exercise aiming at hypertrophy and reviewing the related scientific literature. The derived aspects of relevance for the design of hypertrophy-related training plans are:

1. Screening for individuals at increased risk for adverse exercise-related events, such as those related to cardiovascular, pulmonary, metabolic, and other diseases [22].
2. Definition of a goal [18].
3. Definition of a reliable and valid testing procedure to assess initial performance status. This procedure should derive individual training variables, such as years of resistance training, body composition, previous training volume, training weights, and define training effects, including performance, physiological, subjective, biomechanical or cognitive measures [18, 22, 23].
4. Use of training principles to evaluate the principle of specificity (e.g., exercises selected to achieve a specific goal), the principle of progressive overload (e.g., increasing intensity, load, repetitions, or volume over time), the principle of variation (e.g., changing exercises, repetition ranges, training intensities over time), and the principle of recovery (e.g., ensuring adequate rest between training days or between training the same muscle group) [18, 24].
5. Definition of basic strength training aspects including, but not limited to exercise selection, exercise order, and exercise technique (e.g., regarding safety aspects), as well as training variables like frequency, intensity, and volume [6, 18, 25, 26, 27, 33].

In addition to general training-related aspects, advanced aspects may be considered when prescribing (evidence-based) training plans, such as:

1. Use of advanced exercise methods like the manipulation of movement speed, range of motion or kinematics. Furthermore, time under tension can be manipulated as well as the set endpoint (e.g., ratings of perceived exertion [RPE], reps in reserve [RIR], proximity to failure) [26, 27, 28].
2. Use of advanced unconventional training methods (e.g., drop sets, rest-pause training, or pre-exhaustion), or the equipment used (e.g., blood flow restriction bandages) [6, 18].
3. Application of advanced recovery strategies (e.g., heat therapy, cooling, sleep) [18].
4. Application of nutritional aspects (e.g., micro-/macronutrient intake, hydration) [18, 29].

### Definition of information input into publicly available LLMs

For our study, we selected GPT-4 (accessed via Microsoft Copilot) and Google Gemini (1.0 Pro), which we used on February 15, 2024. These LLMs have rarely been investigated, but since they are available to the public for free, they are likely to be widely used in various everyday use cases.

LLMs, due to their chatbot nature, will encounter diverse inputs from individuals seeking hypertrophy-related training plans, leading to the development of two input scenarios based on factors like prior knowledge and personal experience. We have reported the prompts as entered in the LLMs, including little information (prompt 1) and detailed information (prompt 2) with an additional training plan to provide information about previous training habits (Table 1).

1. “Please provide me with a resistance training plan to increase muscle hypertrophy.”
2. “Please provide me with a resistance training plan to increase muscle hypertrophy over the next 16 weeks. I am a 25-year-old male and have been doing resistance training 4 times a week for the past 8 years. Previous resistance training sessions have lasted 90 minutes. I have access to free weights and machines, both of which I would like to use. I also have training equipment such as belts, straps, bandages that I can use, and I have a body composition scale for monitoring purposes. My body weight is 80 kg with 12% body fat. I am 180 cm tall. I would like to increase the frequency to 5–6 times a week. I want to increase my total muscle mass as much as possible, although I am at an advanced level. I want to emphasize my arms as they are proportionally smaller than the rest of my body. I like to train with 3 seconds long eccentric actions, while the concentric action is explosive. My one-rep maximums in the squat, bench press, and deadlift are 200 kg, 140 kg, and 230 kg respectively. Overall, I want to incorporate advanced training strategies such as drop sets because I enjoy them. I also want to focus on my nutrition and recovery for muscle hypertrophy.”

**TABLE 1:** Previous resistance training plan provided in prompt 2 (inserted February 15, 2024)

Day 1 & Day 3: Lower body & core				
Exercises	Sets	Repetitions	Intensity	Rest periods
Squats	4	8	3 reps in reserve	3 min.
Leg Press	3	10	3 reps in reserve	90 s
Leg ext.	3	15–20	1–2 reps in reserve	90 s
Calf raises	3	15	1–2 reps in reserve	90 s
Weighted sit ups	2	15–20	1–2 reps in reserve	90 s

**Day 2 & Day 4: Upper body**				
**Exercises**	**Sets**	**Repetitions**	**Intensity**	**Rest periods**

Bench press	4	8	3 reps in reserve	3 min.
Rows	4	8	3 reps in reserve	3 min.
Incline bench press	3	10	3 reps in reserve	3 min.
Pulldown	3	12	3 reps in reserve	3 min.
Lateral raises	2	15–20	1–2 reps in reserve	90 s
Biceps curls	3	15	1–2 reps in reserve	90 s
Triceps ext.	3	15	1–2 reps in reserve	90 s

Two authors (TH and LM) independently inserted each prompt into each LLM on the same day to investigate the reliability of each LLM (2/15/2024). This resulted in a total of 8 weekly training plans generated by the LLMs. Among them were two plans created by Google Gemini using little information about a fictitious person provided by two different researchers (referred to as GGL1 and GGL2). Another two plans were generated by Google Gemini based on detailed information about the same fictitious person (referred to as GGD1 and GGD2). A similar approach was used with GPT-4 (accessed via Microsoft Copilot), producing two plans with little information (GPT-4L1 and GPT-4L2) and two more with detailed information (GPT-4D1 and GPT-4D2). The conversation with both LLMs is available in the Appendix (S-Table 1–8).

### Coaching experts

The evaluation of the Google Gemini and GPT-4 derived training plans followed the procedure of previously published studies [16, 19, 20, 21]. Experienced coaches evaluated the provided resistance training plans, focusing on key aspects essential for effective training plan design, as outlined in Table 2. Each aspect was rated on a 1–5 Likert scale. To evaluate the training plans, each coach was required to have at least a bachelor’s degree in sport science and 3 years of coaching experience in strength and conditioning or the field of resistance training. The study was approved by the Ethics Committee of the Faculty of Exercise Science and Training at the University of Würzburg (EV2023/7-2609) and conducted in accordance with the Declaration of Helsinki. Coaches gave informed consent to participate in the study.

**TABLE 2:** Relevant aspects when designing a training plan and corresponding rating scale which was used to evaluate Google Gemini and GPT-4 generated training plans

Relevant aspects when designing a training plan		Rating (1–5 Likert Scale)						
		1 (bad)	2	3	4	5 (good)	Not Applicable	Comment
**General Aspects**	Overall training plan							

	Health screening							

	Defined goal							

	Overall testing procedure							

	Testing procedure regarding initial performance status							

	Testing procedure regarding assessment of individual training variables							

	Testing procedure regarding assessment of training effects							

	Overall monitoring procedure							

**Training principles**	Principle of specificity							

	Principle of progressive overload							

	Principle of variation							

	Principle of recovery							

**Basic strength training aspects**	Exercise selection							

	Exercise order							

	Weekly training frequency per muscle							

	Training intensity per exercise							

	Repetition range per exercise							

	Overall training volume							

	Number of sets per muscle per week							

	Rest periods							

	Exercise technique							

**Advanced training aspects**	Advanced exercise methods							

	Time under tension							

	Set endpoint							

	Advanced training methods							

	Equipment							

	Recovery strategies							

	Nutrition							

### Statistical analysis

As previously performed [16], we calculated descriptive statistics (i.e., median, range) for the Likert scores of all rated items for each question. We tested for normal distribution using the Shapiro-Wilk test. Since the majority of our variables were not normally distributed, we performed a Friedmann ANOVA with Bonferroni correction. The significance level was set at p < 0.05. Fleiss’ Kappa was calculated to assess inter-rater reliability [30]. SPSS (IBM, version 28.0.1.1) was used for all statistical analyses.

## RESULTS

A total of 12 coaching experts (age span: 23–49 years; 4 with a PhD, 5 with a Master’s degree, and 3 with a Bachelor’s degree in Sport Science) with coaching experience of 11.3 ± 5.7 years in resistance training, participated in our study.

For Google Gemini, Fleiss’ Kappa was 0.188 for GGL1, 0.100 for GGL2, 0.139 for GGD1, and 0.121 for GGD2. For GPT-4, Fleiss’ Kappa was 0.046 for GPT-4L1, 0.216 for GPT-4L2, 0.140 for GPT-4D1, and 0.1785 for GPT-4D2. Likert scale charts of each training plan are illustrated in the supplementary material (S-Figure 1–8).

## Reproducibility of LLM output following the same prompt input

Descriptive statistics and results of significance testing for reproducibility between the same prompts within the LLMs are presented in Table 3.

**TABLE 3:** Descriptive analysis (median and range) and results of the significance testing of AI repeatability comparing different training plans generated by Google Gemini and GPT-4. Likert-Scale Ratings were from 1 (“bad”) to 5 (“good”) with 0 indicating “not applicable”.

Relevant aspects when designing a training plan		Median (Range)								Significance testing (same prompt, same LLM) – Repeatability (p-value)			
		GGL1	GGL2	GPT-4L1	GPT-4L2	GGD1	GGD2	GPT-4D1	GPT-4D2	GGL1 vs GGL2	GPT-4L1 vs GPT-4L2	GGD1 vs GGD2	GPT-4D1 vs GPT-4D2
**General Aspects**	Overall training plan	2 (2)	0 (3)	3 (2)	3 (4)	3 (3)	3 (3)	4 (3)	3.5 (4)	1.000	1.000	1.000	1.000

	Health screening	2 (5)	2.5 (5)	0 (3)	2 (5)	1.5 (5)	0.5 (5)	0 (4)	0 (5)	1.000	1.000	1.000	1.000

	Defined goal	2 (4)	0.5 (5)	2 (4)	3.5 (5)	4 (5)	3 (5)	4 (5)	4 (5)	1.000	1.000	1.000	1.000

	Overall testing procedure	0 (4)	0 (2)	0 (2)	0 (3)	0 (4)	0 (4)	0 (5)	0 (5)	1.000	1.000	1.000	1.000

	Testing procedure regarding initial performance status	0 (4)	0 (2)	0 (5)	0 (4)	0 (5)	0 (5)	0 (5)	0 (5)	1.000	1.000	1.000	1.000

	Testing procedure regarding assessment of individual training variables	0 (4)	0 (2)	0 (4)	0 (4)	0 (4)	0 (4)	0 (5)	0 (5)	1.000	1.000	1.000	1.000

	Testing procedure regarding assessment of training effects	0 (4)	0 (2)	0 (5)	0 (3)	0 (4)	0.5 (4)	0 (5)	0 (4)	1.000	1.000	1.000	1.000

	Overall monitoring procedure	0 (3)	0 (2)	0 (4)	0 (3)	0 (4)	0.5 (4)	0.5 (5)	0 (5)	1.000	1.000	1.000	0.940

**Summary Rating**	**< 3**	**n = 8**	**n = 8**	**n = 7**	**n = 6**	**n = 6**	**n = 6**	**n = 6**	**n = 6**				

	**3**	**n = 0**	**n = 0**	**n = 1**	**n = 1**	**n = 2**	**n = 2**	**n = 0**	**n = 0**				

	**> 3**	**n = 0**	**n = 0**	**n = 0**	**n = 1**	**n = 0**	**n = 0**	**n = 2**	**n = 2**				

**Training principles**	Principle of specificity	2 (4)	2 (4)	3 (5)	4 (5)	4 (4)	4 (4)	5 (4)	4 (3)	1.000	1.000	1.000	1.000

	Principle of progressive overload	2.5 (2)	3 (5)	4 (3)	3 (2)	4 (5)	4 (2)	3 (5)	4 (5)	1.000	1.000	1.000	0.762

	Principle of variation	2 (4)	0 (3)	3 (5)	3 (5)	3.5 (5)	4 (5)	4 (5)	4 (5)	1.000	1.000	1.000	1.000

	Principle of recovery	2.5 (4)	3 (5)	3 (3)	3 (5)	3.5 (4)	3.5 (3)	4 (4)	4 (3)	1.000	1.000	1.000	1.000

**Summary Rating**	**< 3**	**n = 4**	**n = 2**	**n = 0**	**n = 0**	**n = 0**	**n = 0**	**n = 0**	**n = 0**				

	**3**	**n = 0**	**n = 2**	**n = 3**	**n = 3**	**n = 0**	**n = 0**	**n = 1**	**n = 0**				

	**> 3**	**n = 0**	**n = 0**	**n = 1**	**n = 1**	**n = 4**	**n = 4**	**n = 3**	**n = 4**				

**Basic strength training aspects**	Exercise selection	2 (3)	0 (4)	4 (3)	4 (4)	4 (5)	4 (3)	4 (3)	4 (3)	1.000	1.000	1.000	1.000

	Exercise order	3.5 (4)	0 (2)	4 (4)	4 (2)	3.5 (4)	4 (3)	4 (4)	4 (4)	0.087	1.000	0.491	1.000

	Weekly training frequency per muscle	2.5 (2)	2 (5)	3 (3)	3 (3)	4 (5)	4 (3)	4 (3)	4 (3)	1.000	1.000	1.000	1.000

	Training intensity per exercise	3 (5)	3 (4)	3 (3)	3 (5)	2 (4)	2 (5)	4 (3)	2.5 (5)	1.000	1.000	1.000	0.129

	Repetition range per exercise	3 (4)	3 (4)	3 (3)	4 (4)	2.5 (3)	3 (3)	4.5 (4)	4 (4)	1.000	1.000	1.000	1.000

	Overall training volume	3 (3)	0 (3)	3 (3)	4 (4)	3 (4)	4 (3)	3 (4)	4 (4)	0.762	1.000	1.000	1.000

	Number of sets per muscle per week	2.5 (3)	0 (5)	3 (2)	3 (3)	3 (4)	4 (3)	3.5 (4)	4 (3)	1.000	1.000	1.000	1.000

	Rest periods	2 (5)	0 (2)	3.5 (3)	4 (4)	3 (3)	3 (4)	4 (4)	4 (4)	0.113	1.000	1.000	1.000

	Exercise technique	1 (4)	0 (3)	0 (3)	0 (4)	0 (4)	0 (4)	0.5 (5)	1 (4)	1.000	1.000	1.000	1.000

**Summary Rating**	**< 3**	**n = 5**	**n = 7**	**n = 1**	**n = 1**	**n = 3**	**n = 2**	**n = 1**	**n = 2**				

	**3**	**n = 3**	**n = 2**	**n = 5**	**n = 3**	**n = 3**	**n = 2**	**n = 1**	**n = 0**				

	**> 3**	**n = 1**	**n = 0**	**n = 3**	**n = 5**	**n = 3**	**n = 5**	**n = 7**	**n = 7**				

**Advanced training aspects**	Advanced exercise methods	0 (1)	0 (2)	0 (1)	0 (3)	3.5 (5)	0 (4)	3 (5)	0 (5)	1.000	1.000	0.189	1.000

	Time under tension	0 (2)	0 (2)	0 (3)	0 (3)	2.5 (5)	0 (4)	1 (5)	0 (5)	1.000	1.000	1.000	1.000

	Set endpoint	1 (5)	0 (4)	0 (4)	0 (4)	0 (4)	0.5 (4)	5 (3)	0 (5)	1.000	1.000	0.762	**0.004**

	Advanced training methods	0 (1)	0 (2)	0 (3)	0.5 (3)	3 (2)	0.5 (5)	4 (4)	4 (3)	1.000	1.000	1.000	1.000

	Equipment	0 (1)	0 (2)	0 (1)	0 (2)	0 (4)	0 (4)	0 (4)	0 (5)	1.000	1.000	1.000	1.000

	Recovery strategies	0 (2)	0 (2)	0 (2)	0 (2)	3.5 (4)	2.5 (4)	4 (3)	4 (5)	1.000	1.000	1.000	1.000

	Nutrition	0 (1)	0 (2)	1.5 (3)	0 (2)	3.5 (4)	2 (4)	4 (4)	4 (4)	1.000	1.000	1.000	1.000

**Summary Rating**	**< 3**	**n = 7**	**n = 7**	**n = 7**	**n = 7**	**n = 3**	**n = 7**	**n = 2**	**n = 4**				

	**3**	**n = 0**	**n = 0**	**n = 0**	**n = 0**	**n = 1**	**n = 0**	**n = 1**	**n = 0**				

	**> 3**	**n = 0**	**n = 0**	**n = 0**	**n = 0**	**n = 3**	**n = 0**	**n = 4**	**n = 3**				

GGL1 = Google Gemini, little information, first try; GGL2 = Google Gemini, little information, second try; GPT-4L1 = GPT-4, little information, first try; GPT-4L2 = GPT-4, little information, second try; GGD1 = Google Gemini, detailed information, first try; GGD2 = Gemini, detailed information, second try; GPT-4D1 = GPT-4, detailed information, first try; GPT-4D2 = GPT-4, detailed information, second try.

## Differences between Google Gemini and GPT-4

Descriptive statistics and results of significance testing between Google Gemini and GPT-4 with different input information are presented in Table 4.

**TABLE 4:** Descriptive analysis (median and range) and results of the significance testing of different AIs comparing different training plans generated by Google Gemini and GPT-4. Likert-Scale Ratings were from 1 (“bad”) to 5 (“good”) with 0 indicating “not applicable”.

Relevant aspects when designing a training plan		Median (Range)								Significance testing (p-value) (same prompt, different LLM)							

		GGL1	GGL2	GPT-4L1	GPT-4L2	GGD1	GGD2	GPT-4D1	GPT-4D2	GGL1 vs GPT-4L1	GGL2 vs GPT-4L2	GGD2 vs GPT-4D2	GGL1 vs GPT-4L2	GGL2 vs GPT-4L1	GGD1 vs GPT-4D1	GGD2 vs GPT-4D1	GGD1 vs GPT-4D2
**General Aspects**	Overall training plan	2 (2)	0 (3)	3 (2)	3 (4)	3 (3)	3 (3)	4 (3)	3.5 (4)	1.000	**0.011**	1.000	0.491	0.439	1.000	1.000	1.000

	Healths creening	2 (5)	2.5 (5)	0 (3)	2 (5)	1.5 (5)	0.5 (5)	0 (4)	0 (5)	1.000	1.000	1.000	1.000	1.000	1.000	1.000	1.000

	Defined goal	2 (4)	0.5 (5)	2 (4)	3.5 (5)	4 (5)	3 (5)	4 (5)	4 (5)	1.000	1.000	1.000	1.000	1.000	1.000	1.000	1.000

	Overall testing procedure	0 (4)	0 (2)	0 (2)	0 (3)	0 (4)	0 (4)	0 (5)	0 (5)	1.000	1.000	1.000	1.000	1.000	1.000	1.000	1.000

	Testing procedure regarding initial performance status	0 (4)	0 (2)	0 (5)	0 (4)	0 (5)	0 (5)	0 (5)	0 (5)	1.000	1.000	1.000	1.000	1.000	1.000	1.000	1.000

	Testing procedure regarding assessment of training effects	0 (4)	0 (2)	0 (4)	0 (4)	0 (4)	0 (4)	0 (5)	0 (5)	1.000	1.000	1.000	1.000	1.000	1.000	1.000	1.000

	Testing procedure regarding assessment of individual training variables	0 (4)	0 (2)	0 (5)	0 (3)	0 (4)	0.5 (4)	0 (5)	0 (4)	1.000	1.000	1.000	1.000	1.000	1.000	1.000	1.000

	Overall monitoring procedure	0 (3)	0 (2)	0 (4)	0 (3)	0 (4)	0.5 (4)	0.5 (5)	0 (5)	1.000	1.000	1.000	1.000	1.000	1.000	1.000	1.000

**Summary Rating**	**<3**	**n = 8**	**n = 8**	**n = 7**	**n = 6**	**n = 6**	**n = 6**	**n = 6**	**n = 6**

	**3**	**n = 0**	**n = 0**	**n = 1**	**n = 1**	**n = 2**	**n = 2**	**n = 0**	**n = 0**

	**>3**	**n = 0**	**n = 0**	**n = 0**	**n = 1**	**n = 0**	**n = 0**	**n = 2**	**n = 2**

**Training principles**	Principle of specificity	2 (4)	2 (4)	3 (5)	4 (5)	4 (4)	4 (4)	5 (4)	4 (3)	1.000	0.129	1.000	0.309	1.000	1.000	1.000	1.000

	Principle of progressive overload	2.5 (2)	3 (5)	4 (3)	3 (2)	4 (5)	4 (2)	3 (5)	4 (5)	1.000	1.000	1.000	1.000	1.000	0.847	1.000	1.000

	Principle of variation	2 (4)	0 (3)	3 (5)	3 (5)	3.5 (5)	4 (5)	4 (5)	4 (5)	1.000	0.685	1.000	1.000	1.000	1.000	1.000	1.000

	Principle of recovery	2.5 (4)	3 (5)	3 (3)	3 (5)	3.5 (4)	3.5 (3)	4 (4)	4 (3)	1.000	1.000	1.000	1.000	1.000	1.000	1.000	1.000

**Summary Rating**	**<3**	**n = 4**	**n = 2**	**n = 0**	**n = 0**	**n = 0**	**n = 0**	**n = 0**	**n = 0**

	**3**	**n = 0**	**n = 2**	**n = 3**	**n = 3**	**n = 0**	**n = 0**	**n = 1**	**n = 0**

	**>3**	**n = 0**	**n = 0**	**n = 1**	**n = 1**	**n = 4**	**n = 4**	**n = 3**	**n = 4**

**Basic strength training aspects**	Exercise selection	2 (3)	0 (4)	4 (3)	4 (4)	4 (5)	4 (3)	4 (3)	4 (3)	0.066	**0.001**	1.000	**0.032**	1.000	1.000	1.000	1.000

	Exercise order	3.5 (4)	0 (2)	4 (4)	4 (2)	3.5 (4)	4 (3)	4 (4)	4 (4)	1.000	**0.000**	1.000	1.000	**0.000**	1.000	1.000	1.000

	Weekly training frequency per muscle	2.5 (2)	2 (5)	3 (3)	3 (3)	4 (5)	4 (3)	4 (3)	4 (3)	1.000	1.000	1.000	1.000	1.000	1.000	1.000	1.000

	Training intensity per exercise	3 (5)	3 (4)	3 (3)	3 (5)	2 (4)	2 (5)	4 (3)	2.5 (5)	1.000	1.000	1.000	1.000	1.000	**0.018**	**0.032**	1.000

	Repetition range per exercise	3 (4)	3 (4)	3 (3)	4 (4)	2.5 (3)	3 (3)	4.5 (4)	4 (4)	1.000	0.309	1.000	1.000	1.000	**0.05**	1.000	1.000

	Overall training volume	3 (3)	0 (3)	3 (3)	4 (4)	3 (4)	4 (3)	3 (4)	4 (4)	1.000	**0.007**	1.000	1.000	0.309	1.000	1.000	1.000

	Number of sets per muscle per week	2.5 (3)	0 (5)	3 (2)	3 (3)	3 (4)	4 (3)	3.5 (4)	4 (3)	1.000	0.099	1.000	1.000	1.000	1.000	1.000	1.000

	Rest periods	2 (5)	0 (2)	3.5 (3)	4 (4)	3 (3)	3 (4)	4 (4)	4 (4)	1.000	**0.002**	1.000	1.000	**0.043**	1.000	1.000	1.000

	Exercise technique	1 (4)	0 (3)	0 (3)	0 (4)	0 (4)	0 (4)	0.5 (5)	1 (4)	1.000	1.000	1.000	1.000	1.000	1.000	1.000	1.000

**Summary Rating**	**<3**	**n = 5**	**n = 7**	**n = 1**	**n = 1**	**n = 3**	**n = 2**	**n = 1**	**n = 2**

	**3**	**n = 3**	**n = 2**	**n = 5**	**n = 3**	**n = 3**	**n = 2**	**n = 1**	**n = 0**

	**>3**	**n = 1**	**n = 0**	**n = 3**	**n = 5**	**n = 3**	**n = 5**	**n = 7**	**n = 7**

**Advanced training aspects**	Advanced exercise methods	0 (1)	0 (2)	0 (1)	0 (3)	3.5 (5)	0 (4)	3 (5)	0 (5)	1.000	1.000	1.000	1.000	1.000	1.000	1.000	1.000

	Time under tension	0 (2)	0 (2)	0 (3)	0 (3)	2.5 (5)	0 (4)	1 (5)	0 (5)	1.000	1.000	1.000	1.000	1.000	1.000	1.000	1.000

	Set endpoint	1 (5)	0 (4)	0 (4)	0 (4)	0 (4)	0.5 (4)	5 (3)	0 (5)	1.000	1.000	1.000	1.000	1.000	0.002	0.015	1.000

	Advanced training methods	0 (1)	0 (2)	0 (3)	0.5 (3)	3 (2)	0.5 (5)	4 (4)	4 (3)	1.000	1.000	0.066	1.000	1.000	1.000	0.043	1.000

	Equipment	0 (1)	0 (2)	0 (1)	0 (2)	0 (4)	0 (4)	0 (4)	0 (5)	1.000	1.000	1.000	1.000	1.000	1.000	1.000	1.000

	Recovery strategies	0 (2)	0 (2)	0 (2)	0 (2)	3.5 (4)	2.5 (4)	4 (3)	4 (5)	1.000	1.000	1.000	1.000	1.000	1.000	0.243	1.000

	Nutrition	0 (1)	0 (2)	1.5 (3)	0 (2)	3.5 (4)	2 (4)	4 (4)	4 (4)	1.000	1.000	1.000	1.000	1.000	1.000	1.000	1.000

**Summary Rating**	**<3**	**n = 7**	**n = 7**	**n = 7**	**n = 7**	**n = 3**	**n = 7**	**n = 2**	**n = 4**

	**3**	**n = 0**	**n = 0**	**n = 0**	**n = 0**	**n = 1**	**n = 0**	**n = 1**	**n = 0**

	**>3**	**n = 0**	**n = 0**	**n = 0**	**n = 0**	**n = 3**	**n = 0**	**n = 4**	**n = 3**

GGL1 = Google Gemini, little information, first try; GGL2 = Google Gemini, little information, secondtry; GPT-4L1 = GPT-4, little information, first try; GPT-4L2 = GPT-4, little information, second try; GGD1 = Google Gemini, detailed information, first try; GGD2 = Gemini, detailed information, secondtry; GPT-4D1 = GPT-4, detailed information, first try; GPT - 4D2 = GPT - 4, detailed information, second try.

### Differences in prompt information density (little information versus detailed information)

Descriptive statistics and results of significance tests between Google Gemini and GPT-4 are presented in Table 5. All other statistical comparisons between LLMs and prompt information density that are not representative of the results presented here (e.g., GGL1 versus GPT-4D2) can be found in the Appendix (S-Table 9).

**TABLE 5:** Descriptive analysis (median and range) and results of the significance testing of different Prompts comparing different training plans generated by Google Gemini and GPT-4. Likert-Scale Ratings were from 1 (“bad”) to 5 (“good”) with 0 indicating “not applicable”.

Relevant aspects when designing a training plan		Median (Range)								Significance testing (p-value) (same prompt, different LLM)							

		GGL1	GGL2	GPT-4L1	GPT-4L2	GGD1	GGD2	GPT-4D1	GPT-4D2	GGL1 vs GPT-4L1	GGL2 vs GPT-4L2	GGD2 vs GPT-4D2	GGL1 vs GPT-4L2	GGL2 vs GPT-4L1	GGD1 vs GPT-4D1	GGD2 vs GPT-4D1	GGD1 vs GPT-4D2
**General Aspects**	Over all training plan	2 (2)	0 (3)	3 (2)	3 (4)	3 (3)	3 (3)	4 (3)	3.5 (4)	1.000	0.018	0.685	1.000	0.129	0.685	1.000	1.000

	Health screening	2 (5)	2.5 (5)	0 (3)	2 (5)	1.5 (5)	0.5 (5)	0 (4)	0 (5)	1.000	1.000	1.000	1.000	1.000	1.000	1.000	1.000

	Defined goal	2 (4)	0.5 (5)	2 (4)	3.5 (5)	4 (5)	3 (5)	4 (5)	4 (5)	0.309	1.000	0.024	1.000	1.000	1.000	0.087	1.000

	Overall testing procedure	0 (4)	0 (2)	0 (2)	0 (3)	0 (4)	0 (4)	0 (5)	0 (5)	1.000	1.000	1.000	1.000	1.000	1.000	1.000	1.000

	Testing procedure regarding initial performance status	0 (4)	0 (2)	0 (5)	0 (4)	0 (5)	0 (5)	0 (5)	0 (5)	1.000	1.000	1.000	1.000	1.000	1.000	1.000	1.000

	Testing procedure regarding assessment of individual training variables	0 (4)	0 (2)	0 (4)	0 (4)	0 (4)	0 (4)	0 (5)	0 (5)	1.000	1.000	1.000	1.000	1.000	1.000	1.000	1.000

	Testing procedure regarding assessment of training effects	0 (4)	0 (2)	0 (5)	0 (3)	0 (4)	0.5 (4)	0 (5)	0 (4)	1.000	1.000	1.000	1.000	1.000	1.000	1.000	1.000

	Overall monitoring procedure	0 (3)	0 (2)	0 (4)	0 (3)	0 (4)	0.5 (4)	0.5 (5)	0 (5)	1.000	1.000	1.000	1.000	1.000	1.000	1.000	1.000

**Summary Rating**	<3	**n = 8**	**n = 8**	**n = 7**	**n = 6**	**n = 6**	**n = 6**	**n = 6**	**n = 6**								

	**3**	**n = 0**	**n = 0**	**n = 1**	**n = 1**	**n = 2**	**n = 2**	**n = 0**	**n = 0**								

	**>3**	**n = 0**	**n = 0**	**n = 0**	**n = 1**	**n = 0**	**n = 0**	**n = 2**	**n = 2**								

**Training principles**	Principle of specificity	2 (4)	2 (4)	3 (5)	4 (5)	4 (4)	4 (4)	5 (4)	4 (3)	0.550	0.129	0.057	1.000	0.243	0.309	1.000	1.000

	Principle of progressive overload	2.5 (2)	3 (5)	4 (3)	3 (2)	4 (5)	4 (2)	3 (5)	4 (5)	0.113	1.000	1.000	1.000	1.000	1.000	1.000	1.000

	Principle of variation	2 (4)	0 (3)	3 (5)	3 (5)	3.5 (5)	4 (5)	4 (5)	4 (5)	0.847	0.037	1.000	1.000	0.066	0.550	0.491	1.000

	Principle of recovery	2.5 (4)	3 (5)	3 (3)	3 (5)	3.5 (4)	3.5 (3)	4 (4)	4 (3)	0.940	1.000	1.000	1.000	1.000	1.000	1.000	1.000

**Summary Rating**	<3	**n = 4**	**n = 2**	**n = 0**	**n = 0**	**n = 0**	**n = 0**	**n = 0**	**n = 0**								

	**3**	**n = 0**	**n = 2**	**n = 3**	**n = 3**	**n = 0**	**n = 0**	**n = 1**	**n = 0**								

	**>3**	**n = 0**	**n = 0**	**n = 1**	**n = 1**	**n = 4**	**n = 4**	**n = 3**	**n = 4**								

**Basic strength training aspects**	Exercise selection	2 (3)	0 (4)	4 (3)	4 (4)	4 (5)	4 (3)	4 (3)	4 (3)	1.000	0.018	1.000	1.000	0.685	0.309	1.000	1.000

	Exercise order	3.5 (4)	0 (2)	4 (4)	4 (2)	3.5 (4)	4 (3)	4 (4)	4 (4)	1.000	0.000	1.000	1.000	1.000	1.000	1.000	1.000

	Weekly training frequency permuscle	2.5 (2)	2 (5)	3 (3)	3 (3)	4 (5)	4 (3)	4 (3)	4 (3)	1.000	0.274	0.940	1.000	1.000	0.391	0.847	1.000

	Training intensity per exercise	3 (5)	3 (4)	3 (3)	3 (5)	2 (4)	2 (5)	4 (3)	2.5 (5)	1.000	1.000	1.000	1.000	1.000	1.000	1.000	0.274

	Repetition range per exercise	3 (4)	3 (4)	3 (3)	4 (4)	2.5 (3)	3 (3)	4.5 (4)	4 (4)	1.000	0.762	1.000	1.000	1.000	1.000	1.000	1.000

	Overall training volume	3 (3)	0 (3)	3 (3)	4 (4)	3 (4)	4 (3)	3 (4)	4 (4)	1.000	0.006	1.000	1.000	0.391	1.000	1.000	1.000

	Number of sets per muscle per week	2.5 (3)	0 (5)	3 (2)	3 (3)	3 (4)	4 (3)	3.5 (4)	4 (3)	1.000	0.099	1.000	1.000	0.309	1.000	1.000	1.000

	Rest periods	2 (5)	0 (2)	3.5 (3)	4 (4)	3 (3)	3 (4)	4 (4)	4 (4)	1.000	0.076	1.000	1.000	0.003	1.000	1.000	1.000

	Exercise technique	1 (4)	0 (3)	0 (3)	0 (4)	0 (4)	0 (4)	0.5 (5)	1 (4)	1.000	1.000	1.000	1.000	1.000	1.000	1.000	1.000

**Summary Rating**	**<3**	**n = 5**	**n = 7**	**n = 1**	**n = 1**	**n = 3**	**n = 2**	**n = 1**	**n = 2**								

	**3**	**n = 3**	**n = 2**	**n = 5**	**n = 3**	**n = 3**	**n = 2**	**n = 1**	**n = 0**								

	**>3**	**n = 1**	**n = 0**	**n = 3**	**n = 5**	**n = 3**	**n = 5**	**n = 7**	**n = 7**								

**Advanced training aspects**	Advanced exercise methods	0 (1)	0 (2)	0 (1)	0 (3)	3.5 (5)	0 (4)	3 (5)	0 (5)	0.024	1.000	0.309	1.000	0.015	1.000	1.000	1.000

	Time under tension	0 (2)	0 (2)	0 (3)	0 (3)	2.5 (5)	0 (4)	1 (5)	0 (5)	0.550	1.000	1.000	1.000	0.309	1.000	1.000	1.000

	Set end point	1 (5)	0 (4)	0 (4)	0 (4)	0 (4)	0.5 (4)	5 (3)	0 (5)	1.000	1.000	0.000	1.000	1.000	1.000	1.000	0.004

	Advanced training methods	0 (1)	0 (2)	0 (3)	0.5 (3)	3 (2)	0.5 (5)	4 (4)	4 (3)	0.007	1.000	0.001	0.028	0.013	1.000	0.001	0.018

	Equipment	0 (1)	0 (2)	0 (1)	0 (2)	0 (4)	0 (4)	0 (4)	0 (5)	1.000	1.000	1.000	1.000	1.000	1.000	1.000	1.000

	Recovery strategies	0 (2)	0 (2)	0 (2)	0 (2)	3.5 (4)	2.5 (4)	4 (3)	4 (5)	0.015	0.762	0.000	0.002	0.015	0.762	0.007	0.000

	Nutrition	0 (1)	0 (2)	1.5 (3)	0 (2)	3.5 (4)	2 (4)	4 (4)	4 (4)	0.011	0.147	0.009	0.000	0.021	0.087	0.013	0.000

**Summary Rating**	**<3**	**n = 7**	**n = 7**	**n = 7**	**n = 7**	**n = 3**	**n = 7**	**n = 2**	**n = 4**

	**3**	**n = 0**	**n = 0**	**n = 0**	**n = 0**	**n = 1**	**n = 0**	**n = 1**	**n = 0**

	**>3**	**n = 0**	**n = 0**	**n = 0**	**n = 0**	**n = 3**	**n = 0**	**n = 4**	**n = 3**

GGL1 = Google Gemini, little information, first try; GGL2 = Google Gemini, little information, second try; GPT - 4L1 = GPT - 4, little information, first try; GPT - 4L2 = GPT - 4, little information, second try; GGD1 = Google Gemini, detailed information, first try; GGD2 = Gemini, detailed information, secondtry; GPT - 4D1 = GPT - 4, detailed information, first try; GPT - 4D2 = GPT - 4, detailed information, second try.

## DISCUSSION

Our study aimed to investigate the quality of resistance training plans focusing on muscle hypertrophy generated by Google Gemini and GPT-4 accessed via Microsoft Copilot and whether the provided training plans can be generated repeatedly when providing similar prompts multiple times. We report here that when hypertrophy-focused training plans are repeatedly generated by the same LLM (i.e., Google Gemini or GPT-4) using the same prompts, the resulting plans consistently maintain a comparable level of quality as assessed by coaching experts. Moreover, the quality of muscle hypertrophy related training plans generated by GPT-4 was rated higher compared to Google Gemini, irrespective of level of provided input information. Noticeably, the quality of muscle hypertrophy-related training plans increase with more detailed information input.

## Reproducibility of LLM´s

When provided with identical prompts, Google Gemini and GPT-4 generated muscle hypertrophy-related training plans that were rated similarly on the 5-point Likert Scale across 27 of 28 items. The only exception was the “set endpoint” item. In this case, GPT-4D1 had a median rating of 5, whereas GPT-4D2 had a median rating of 0. The set endpoint was identified within the previous resistance training program in GPT-4D1 but not in GPT-4D2. Therefore, it is recommended that users request any missing information by submitting a follow-up prompt (i.e., check-backs) if the initial prompt is insufficient [16].

Despite being rated similarly in quality by the coaching experts, muscle hypertrophy-related training plans differed in their exercise prescriptions and variables (see S-Table 1–8). Athletes and coaches must verify that the recommended exercises are feasible for the individual athlete and can be performed with the available equipment. To the best of our knowledge, this research is the first to assess the reproducibility of the output quality of publicly available LLMs, such as Google Gemini and GPT-4. Consequently, we cannot compare our results to existing literature. However, a recent study investigated ChatGPT’s use as a sample-size calculator for study design development and found that when the same prompt was reused, ChatGPT produced a completely different output [13]. This is partially consistent with our study, although the quality as rated by the coaching experts was similar. As LLMs are rapidly evolving, we encourage further research in this area to investigate the reproducibility of recommendations provided by LLMs.

## Differences in quality of LLMs generated muscle hypertrophyrelated training plans

Our results show that the quality of hypertrophy-related training plans generated by GPT-4 was rated higher compared to Google Gemini (regardless of the level of input information provided) and that for both LLMs, the quality of generated hypertrophy-related training plans increased with more detailed input information.

Our second prompt with little information (“Please provide me with a resistance training plan to increase muscle hypertrophy”; S-Table 2) inserted into Google Gemini did not result in a resistance training plan. Instead, Gemini responded with general principles of resistance training that require further input to generate the appropriate training plan. Although this is not always the case, as shown by our first attempt in Gemini (S-Table 1), it seems necessary to provide sufficient information to the LLM. Furthermore, providing little information often resulted in training prescriptions that were missing (i.e., training intensity) or irrational. For example, using the same prompt as in Google Gemini in GPT-4 resulted in a recommendation to train each muscle group at least twice a week. However, in the training plan itself, muscle groups were trained once a week, indicating inconsistency within the LLM.

Our findings are in line with available literature which states that training plans improve with more input information but are not rated optimally [12, 14, 15, 16, 17, 31].

Washif et al. assessed GPT-3.5 and GPT-4’s ability to create resistance training plans for intermediate and advanced athletes and found some programming variables and recommendations were insufficient (e.g., exercise selection, exercise tempo, contemporary practices) [17]. The selection of exercises was evaluated as moderately sufficient for promoting strength development and hypertrophy, and the authors identified discrepancies in the prescribed exercise tempo (e.g., 2 seconds eccentric phase/0 seconds pause/2 seconds concentric phase [2/0/2]), noting that it was inconsistently applied and did not fully align with contemporary research recommendations, which suggest a medium-paced eccentric phase and a rapid concentric phase (e.g., 3–4/0/1) [32]. Furthermore, time-efficient techniques for promoting strength gains or muscle hypertrophy (e.g., drop sets) were omitted, and an overemphasis on muscle damage as a mechanism for hypertrophy was noted as another limitation [17]. These limitations suggest that while GPT-3.5 and GPT-4 can generate training plans, they may not always align with specific goals. This aligns with our findings, where time under tension, which is important for hypertrophy [26], was often omitted. These shortcomings indicate the need for further refinement of these LLMs, such as GPT or Google Gemini, and emphasize the need for caution in their use.

Other studies also noticed the imperfection of LLMs [12, 14, 15, 31]. For instance, Zaleski et al. used a mixed-methods approach to evaluate the comprehensiveness and accuracy of ChatGPT’s exercise recommendations from open-text queries and found that AI-generated advice was 41.2% comprehensive and 90.7% accurate compared to gold-standard exercise guidelines [31]. Dergaa et al. evaluated GPT-4’s ability to generate exercise prescriptions for five hypothetical patient profiles and concluded that while AI-generated plans offer a safe starting point, they are inadequate for optimizing long-term fitness and health [14]. In addition, previous studies of ChatGPT’s ability to act as a psychiatric provider [12] or nutrition consultant [15] yielded similar results. ChatGPT can provide appropriate information in less complex scenarios. However, as complexity and vulnerability increase, tailored recommendations are inadequate and sometimes dangerous [12].

Therefore, exercise professionals should provide LLMs with detailed information and carefully review LLM-generated recommendations for muscle hypertrophy-related training regimens and not blindly implement them into practice due to the risk of lack of information output

Our research shows that GPT-4 accessed via Microsoft Copilot has higher ratings in exercise selection, exercise order, training intensity, repetition range, training volume, rest periods, and set endpoint compared to Google Gemini. Similarly, it has been reported that GPT-4 outperforms previous versions of GPT (i.e., GPT-3.5) in variables such as ‘sets and repetitions’ and ‘rest intervals’ [17]. Although the existing literature is limited, it might be argued that GPT-4 currently outperforms both its previous versions and Google Gemini in providing recommendations for strength training plans.

## Strength, limitations and future research

We were able to compare two different LLMs, provide them with different input information densities, and assess for the first time the reproducibility of the quality of recommendations provided by LLMs.

Although some LLMs provide better quality resistance training plans than others, caution should be taken when implementing them blindly. It should be noted that while a high-quality hypertrophy-related training plan is important for athletes, other aspects such as explanation of the training program to the athlete, frequent training plan adjustments and the athlete-coach relationship are crucial in the training process. Additionally, athletes may lack evidence-based resistance training knowledge and may be unable to evaluate and adjust inappropriate training recommendations from LLMs. Consequently, a coach is essential in the training process of athletes and cannot currently be replaced by LLMs and their recommendations for muscle hypertrophy, although LLMs can provide a baseline for training recommendations [14, 16, 17]. Since the quality of LLM recommendations depends on the quality of the input, and given their widespread use and increasing availability, it seems prudent for athletes and coaches to be educated about the use, potential and limitations of these forms of AI in order to use them safely.

Our study is not without limitations. Firstly, our research is limited to the versions of GPT-4 and Google Gemini as of February 15, 2024. Because publicly available LLMs are evolving rapidly, new models are continuously being developed. Therefore, future LLMs may be capable of providing high-quality, reference-based, hypertrophy-related resistance training plans. However, in agreement with our work, previous studies have highlighted that LLMs (specifically ChatGPT) can be used as tool for creating initial, context-dependent frameworks in medicine [12], health promotion [14], and exercise science [16, 17]. These frameworks still require the expertise of human specialists to tailor them to individual scenarios, ensuring that users are not put at risk by relying solely on LLMs. As LLM versions continue to evolve, we also highlight the challenge of comparing specific outcomes, such as the quality of training prescriptions, across studies that utilize different versions of LLMs.

This is because updates and changes to the algorithms in newer versions of LLMs could significantly affect their performance and the quality of their outputs. Consequently, we emphasize that studies involving LLMs are relevant only to the specific versions being investigated at the time. We suggest the development of a regulatory framework in sport and exercise science to address the proper use and application of LLMs, as well as methods for comparing their outputs within the field. This framework would help sport practitioners understand how to effectively integrate LLMs into exercise science and apply them appropriately in practice.

It is important to note that the versions of Google Gemini and GPT-4 used in this study include references for generating hypertrophy-related resistance training plans. Caution should be taken regarding the quality and existence of these references, as LLMs can fabricate invented references [34].

We reported low Fleiss Kappa values (Fleiss Kappa = 0.046 to 0.216), indicating low interrater reliability This is consistent with previous work [16] and despite the fact that the coaching experts were well-educated and experienced in the field of exercise science. However, the influence of certain training parameters (e.g., resistance training volume [35, 36]) or novel resistance training trends (e.g., stretch-mediated hypertrophy [37]) on muscle hypertrophy has not been fully elucidated. For example, although research suggests that a resistance training volume of at least 10 sets per muscle group is efficient for maximizing muscle hypertrophy [36], a threshold at which a certain number of sets per week no longer induces “more” muscle hypertrophy is unclear [35]. Thus, coaching experts may have different perspectives on the importance of training aspects related to muscle hypertrophy.

We highlight the investigation of different, novel, and new versions of LLMs in future studies, with particular attention on the comparison of LLM-generated resistance training plans with traditionally designed training plans by certified coaches. Furthermore, it should be stressed that research on female-derived training plans compared to male-derived plans is very scarce and would open up new research opportunities in the field of artificial intelligence. Although previous research with ChatGPT has shown that prompts containing female versus male individuals lead to similar strength training recommendations [17], it is unclear whether this is consistent for other sports or training regimens in different LLMs.

## CONCLUSIONS

Our findings indicate that AI technology (in this case GPT-4 and Google Gemini) can generate muscle hypertrophy-related training plans consistently with similar quality when identical prompts are used with both LLMs concomitantly. We found that the quality of these training plans improves with more detailed prompt information input. Notably, GPT-4 outperformed Google Gemini in quality, regardless of the input detail level. These findings underscore the importance of providing detailed information to LLMs for optimal outcomes. Moreover, LLMs did not always provide sufficient training prescriptions, highlighting the importance of human expertise and experience to manually customize LLM derived training plans. If LLMs are to be used safely in practice to take advantage of their potential benefits in training plan generation, sport professionals need to know what information to enter into LLMs and should carefully check provided training plans.

## Supplementary Material

Reproducibility and quality of hypertrophy-related training plans generated by GPT-4 and Google Gemini as evaluated by coaching experts

## References

[1] Carvalho L, Junior RM, Barreira J, Schoenfeld BJ, Orazem J, Barroso R. Muscle hypertrophy and strength gains after resistance training with different volume-matched loads: a systematic review and meta-analysis. Appl Physiol Nutr Metab. 2022; 47(4):357–68. doi:10.1139/apnm-2021-0515 Cited in: PubMed; PMID . 35015560

[2] Lim C, Nunes EA, Currier BS, McLeod JC, Thomas ACQ, Phillips SM. An Evidence-Based Narrative Review of Mechanisms of Resistance Exercise-Induced Human Skeletal Muscle Hypertrophy. Med Sci Sports Exerc. 2022; 54(9):1546–59. doi:10.1249/MSS.0000000000002929 Cited in: PubMed; PMID . 35389932 PMC9390238

[3] Wackerhage H, Schoenfeld BJ, Hamilton DL, Lehti M, Hulmi JJ. Stimuli and sensors that initiate skeletal muscle hypertrophy following resistance exercise. J Appl Physiol (1985). 2019; 126(1):30–43. doi:10.1152/japplphysiol.00685.2018 Cited in: PubMed; PMID . 30335577

[4] Schoenfeld B. Squatting kinematics and kinetics and their application to exercise performance. J Strength Cond Res. 2010; 24(12):3497–506. doi:10.1519/JSC.0b013e3181bac2d7 Cited in: PubMed; PMID . 20182386

[5] van Every DW, Coleman M, Plotkin DL, Zambrano H, van Hooren B, Larsen S, Nuckols G, Vigotsky AD, Schoenfeld BJ. Biomechanical, Anthropometric and Psychological Determinants of Barbell Bench Press Strength. Sports (Basel). 2022; 10(12). doi: 10.3390/sports10120199 Cited in: PubMed; PMID . 36548496 PMC9785143

[6] Bernárdez-Vázquez R, Raya-González J, Castillo D, Beato M. Resistance Training Variables for Optimization of Muscle Hypertrophy: An Umbrella Review. Front Sports Act Living. 2022; 4949021. doi: 10.3389/fspor.2022.949021 Cited in: PubMed; PMID . 35873210 PMC9302196

[7] Camargo JBB de, Brigatto FA, Zaroni RS, Trindade TB, Germano MD, Junior ACT, Oliveira TP de, Marchetti PH, Prestes J, Lopes CR. Manipulating Resistance Training Variables to Induce Muscle Strength and Hypertrophy: A Brief Narrative Review. Int J Exerc Sci. 2022; 15(4):910–33. Cited in: PubMed; PMID . 36157335 10.70252/VYUB3717 PMC9458289

[8] Schoenfeld B, Fisher J, Grgic J, Haun C, Helms E, Phillips S, Steele J, Vigotsky A. Resistance Training Recommendations to Maximize Muscle Hypertrophy in an Athletic Population: Position Stand of the IUSCA. 1. 2021; 1(1). doi: 10.47206/ijsc.v1i1.81

[9] Deng J, Lin Y. The Benefits and Challenges of ChatGPT: An Overview. FCIS. 2022; 2(2):81–3. doi:10.54097/fcis.v2i2.4465

[10] Dergaa I, Chamari K, Zmijewski P, Ben Saad H. From human writing to artificial intelligence generated text: examining the prospects and potential threats of ChatGPT in academic writing. Biol Sport. 2023; 40(2):615–22. doi: 10.5114/biolsport.2023.125623 Cited in: PubMed; PMID . 37077800 PMC10108763

[11] Thirunavukarasu AJ, Ting DSJ, Elangovan K, Gutierrez L, Tan TF, Ting DSW. Large language models in medicine. Nat Med. 2023; 29(8):1930–40. doi: 10.1038/s41591-023-02448-8 Cited in: PubMed; PMID . 37460753

[12] Dergaa I, Fekih-Romdhane F, Hallit S, Loch AA, Glenn JM, Fessi MS, Ben Aissa M, Souissi N, Guelmami N, Swed S, El Omri A, Bragazzi NL, Ben Saad H. ChatGPT is not ready yet for use in providing mental health assessment and interventions. Front Psychiatry. 2023; 141277756. doi: 10.3389/fpsyt.2023.1277756 Cited in: PubMed; PMID . 38239905 PMC10794665

[13] Methnani J, Latiri I, Dergaa I, Chamari K, Ben Saad H. ChatGPT for Sample-Size Calculation in Sports Medicine and Exercise Sciences: A Cautionary Note. Int J Sports Physiol Perform. 2023; 18(10):1219–23. doi: 10.1123/ijspp.2023-0109 Cited in: PubMed; PMID . 37536678

[14] Dergaa I, Saad HB, El Omri A, Glenn JM, Clark CCT, Washif JA, Guelmami N, Hammouda O, Al-Horani RA, Reynoso-Sánchez LF, Romdhani M, Paineiras-Domingos LL, Vancini RL, Taheri M, Mataruna-Dos-Santos LJ, Trabelsi K, Chtourou H, Zghibi M, Eken Ö, Swed S, Aissa MB, Shawki HH, El-Seedi HR, Mujika I, Seiler S, Zmijewski P, Pyne DB, Knechtle B, Asif IM, Drezner JA, Sandbakk Ø, Chamari K. Using artificial intelligence for exercise prescription in personalised health promotion: A critical evaluation of OpenAI’s GPT-4 model. Biol Sport. 2024; 41(2):221–41. doi: 10.5114/biolsport.2024.133661 Cited in: PubMed; PMID . 38524814 PMC10955739

[15] Dergaa I, Ben Saad H, Ghouili H, M Glenn J, El Omri A, Slim I, Hasni Y, Taheri M, Ben Aissa M, Guelmami N, Al-Horani R, Washif JA, Shoib S, Mohammed Alyasiri O, Jose Mataruna-Dos-Santos L, Ferreira Alves R, Ibrahim Ceylan H, Swed S. Z, Alshahrani N, Chalghaf N, Dai H, Luigi Bragazzi N, Chamari K. Evaluating the Applicability and Appropriateness of ChatGPT as a Source for Tailored Nutrition Advice: A Multi-Scenario Study. NAJM. 2024; 2(1):1–16. doi: 10.61838/kman.najm.2.1.1

[16] Düking P, Sperlich B, Voigt L, van Hooren B, Zanini M, Zinner C. ChatGPT Generated Training Plans for Runners are not Rated Optimal by Coaching Experts, but Increase in Quality with Additional Input Information. Journal of Sports Science & Medicine. 2024; 23(1):56–72. doi: 10.52082/jssm.2024.56 Cited in: PubMed; PMID . 38455449 PMC10915606

[17] Washif JA, Pagaduan J, James C, Dergaa I, Beaven CM. Artificial intelligence in sport: Exploring the potential of using ChatGPT in resistance training prescription. Biol Sport. 2024; 41(2):209–20. doi: 10.5114/biolsport.2024.132987 Cited in: PubMed; PMID . 38524820 PMC10955742

[18] Haff G, Triplett NT, editors. Essentials of strength training and conditioning. Champaign, IL, Windsor, ON, Leeds: Human Kinetics; 2016. 735 p. eng.

[19] Ayers JW, Poliak A, Dredze M, Leas EC, Zhu Z, Kelley JB, Faix DJ, Goodman AM, Longhurst CA, Hogarth M, Smith DM. Comparing Physician and Artificial Intelligence Chatbot Responses to Patient Questions Posted to a Public Social Media Forum. JAMA Intern Med. 2023; 183(6):589–96. doi: 10.1001/jamainternmed.2023.1838 Cited in: PubMed; PMID . 37115527 PMC10148230

[20] Lukac S, Dayan D, Fink V, Leinert E, Hartkopf A, Veselinovic K, Janni W, Rack B, Pfister K, Heitmeir B, Ebner F. Evaluating ChatGPT as an Adjunct for the Multidisciplinary Tumor Board Decision-Making in Primary Breast Cancer Cases; 2023. 10.1007/s00404-023-07130-5 PMC10579162 37458761

[21] Seth I, Cox A, Xie Y, Bulloch G, Hunter-Smith DJ, Rozen WM, Ross RJ. Evaluating Chatbot Efficacy for Answering Frequently Asked Questions in Plastic Surgery: A ChatGPT Case Study Focused on Breast Augmentation. Aesthet Surg J. 2023; 43(10):1126–35. doi: 10.1093/asj/sjad140 Cited in: PubMed; PMID . 37158147

[22] Thompson PD, Arena R, Riebe D, Pescatello LS. ACSM’s new preparticipation health screening recommendations from ACSM’s guidelines for exercise testing and prescription, ninth edition. Curr Sports Med Rep. 2013; 12(4):215–7. doi:10.1249/JSR.0b013e31829a68cf Cited in: PubMed; PMID . 23851406

[23] Jeffries AC, Marcora SM, Coutts AJ, Wallace L, McCall A, Impellizzeri FM. Development of a Revised Conceptual Framework of Physical Training for Use in Research and Practice. Sports Med. 2022; 52(4):709–24. doi: 10.1007/s40279-021-01551-5 Cited in: PubMed; PMID . 34519982

[24] Stone M, Plisk S, Collins D. Training principles: evaluation of modes and methods of resistance training--a coaching perspective. Sports Biomech. 2002; 1(1):79–103. doi: 10.1080/14763140208522788 Cited in: PubMed; PMID . 14658137

[25] Schoenfeld B, Fisher J, Grgic J, Haun C, Helms E, Phillips S, Steele J, Vigotsky A. Resistance Training Recommendations to Maximize Muscle Hypertrophy in an Athletic Population: Position Stand of the IUSCA. 1. 2021; 1(1). doi: 10.47206/ijsc.v1i1.81

[26] Toigo M, Boutellier U. New fundamental resistance exercise determinants of molecular and cellular muscle adaptations. Eur J Appl Physiol. 2006; 97(6):643–63. doi: 10.1007/s00421-006-0238-1 Cited in: PubMed; PMID . 16845551

[27] Coratella G. Appropriate Reporting of Exercise Variables in Resistance Training Protocols: Much more than Load and Number of Repetitions. Sports Med-Open. 2022; 8(1):99. doi:10.1186/s40798-022-00492-1 Cited in: PubMed; PMID . 35907047 PMC9339067

[28] Androulakis Korakakis P, Wolf M, Coleman M, Burke R, Piñero A, Nippard J, Schoenfeld BJ. Optimizing Resistance Training Technique to Maximize Muscle Hypertrophy:A Narrative Review. Journal of Functional Morphology and Kinesiology. 2023; 9(1):9. doi: 10.3390/jfmk9010009 Cited in: PubMed; PMID . 38249086 PMC10801605

[29] Kerksick CM, Arent S, Schoenfeld BJ, Stout JR, Campbell B, Wilborn CD, Taylor L, Kalman D, Smith-Ryan AE, Kreider RB, Willoughby D, Arciero PJ, VanDusseldorp TA, Ormsbee MJ, Wildman R, Greenwood M, Ziegenfuss TN, Aragon AA, Antonio J. International society of sports nutrition position stand: nutrient timing. J Int Soc Sports Nutr. 2017;1433. doi: 10.1186/s12970-017-0189-4 Cited in: PubMed; PMID . 28919842 PMC5596471

[30] Fleiss JL. Measuring nominal scale agreement among many raters. Psychological Bulletin. 1971; 76(5):378–82. doi: 10.1037/h0031619

[31] Zaleski AL, Berkowsky R, Craig KJT, Pescatello LS. Comprehensiveness, Accuracy, and Readability of Exercise Recommendations Provided by an AI-Based Chatbot: Mixed Methods Study. JMIR medical education. 2024; 10e51308. doi: 10.2196/51308 Cited in: PubMed; PMID . 38206661 PMC10811574

[32] Moreno-Villanueva A, Pino-Ortega J, Rico-González M. Effect of Repetition Duration—Total and in Different Muscle Actions—On the Development of Strength, Power, and Muscle Hypertrophy: A Systematic Review. Strength & Conditioning Journal. 2022; 44(5):39–56. doi: 10.1519/SSC.0000000000000695

[33] Schoenfeld BJ, Peterson MD, Ogborn D, Contreras B, Sonmez GT. Effects of Low-vs. High-Load Resistance Training on Muscle Strength and Hypertrophy in Well-Trained Men. J Strength Cond Res. 2015; 29(10):2954–63. doi: 10.1519/JSC.0000000000000958 Cited in: PubMed; PMID . 25853914

[34] Eysenbach G. The Role of ChatGPT, Generative Language Models, and Artificial Intelligence in Medical Education: A Conversation With ChatGPT and a Call for Papers. JMIR medical education. 2023; 9e46885. doi: 10.2196/46885 Cited in: PubMed; PMID . 36863937 PMC10028514

[35] Enes A, Souza EO de, Souza-Junior TP. Effects of Different Weekly Set Progressions on Muscular Adaptations in Trained Males: Is There a Dose-Response Effect? Med Sci Sports Exerc. 2024; 56(3):553–63. doi: 10.1249/MSS.0000000000003317. Cited in: PubMed; PMID . 37796222

[36] Schoenfeld BJ, Ogborn D, Krieger JW. Dose-response relationship between weekly resistance training volume and increases in muscle mass: A systematic review and meta-analysis. J Sports Sci. 2017; 35(11):1073–82. doi: 10.1080/02640414.2016.1210197 Cited in: PubMed; PMID . 27433992

[37] Warneke K, Lohmann LH, Lima CD, Hollander K, Konrad A, Zech A, Nakamura M, Wirth K, Keiner M, Behm DG. Physiology of Stretch-Mediated Hypertrophy and Strength Increases: A Narrative Review. Sports Med. 2023; 53(11):2055–75. doi: 10.1007/s40279-023-01898-x Cited in: PubMed; PMID . 37556026 PMC10587333

