# Supplementary material for: Reproducibility and quality of hypertrophy-related training plans generated by GPT-4 and Google Gemini as evaluated by coaching experts
Source: Biol Sport. 2024 Dec 18;42(2):289–329. doi: 10.5114/biolsport.2025.145911 (PMC11963122; doi:10.5114/biolsport.2025.145911)
Supplement: Reproducibility and quality of hypertrophy-related training plans generated by GPT-4 and Google Gemini as evaluated by coaching experts [file JBS-42-55336-s1.pdf]

S-TABLE 1. Conversation with Google Gemini and little information input (first attempt) on February 15, 2024

Inserted Question by Human	Response Generated by Gemini
**Please provide me with a resistance training plan to increase muscle hypertrophy.**	Unfortunately, I cannot create a personalized resistance training plan for you without further information. Providing a safe and effective plan requires understanding your individual factors, such as:<br>1. Your current fitness level: Are you a beginner, intermediate, or advanced lifter? This will determine the appropriate intensity and volume of your training.<br>2. Your experience with resistance training: Have you lifted weights before, and if so, what kind of program did you follow? This helps me understand your familiarity with different exercises and techniques.<br>3. Your specific goals: Do you want to build muscle mass overall, or focus on specific muscle groups? Do you have any time constraints or limitations?<br>4. Any injuries or medical conditions: It's important to know if you have any conditions that might require modifications to your training program.<br><br>However, I can provide you with some general guidelines for creating a resistance training plan for muscle hypertrophy:<br><br>**General principles:**<br>• Focus on compound exercises: These exercises work multiple muscle groups at the same time, which is more efficient for building muscle mass. Examples include squats, lunges, deadlifts, push-ups, rows, and pull-ups.<br>• Train each muscle group 2–3 times per week: This allows for sufficient rest and recovery between workouts.<br>• Aim for 8–12 repetitions per set with a weight that challenges you for the last few reps. You can adjust the weight and reps over time as you get stronger.<br>• Perform 3–6 sets per exercise.<br>• Rest for 60–90 seconds between sets.<br>• Warm up before each workout and cool down afterwards.<br>• Prioritize proper form over lifting heavy weights.<br>• Ensure adequate sleep and nutrition to support muscle growth.<br><br>**Additional resources:**<br>• The American College of Sports Medicine (ACSM): https://www.acsm.org/<br>• The National Strength and Conditioning Association (NSCA): https://www.nsca.com/<br>• The National Institutes of Health (NIH): https://www.nih.gov/<br><br>Remember, it's important to listen to your body and adjust your training plan as needed. If you are unsure about anything, consult with a certified personal trainer or other qualified healthcare professional.

S-TABLE 1. Continue.

Inserted Question by Human	Response Generated by Gemini
**Follow-up prompt:** I'm new to resistance training, having primarily used machines for the past 6 months. My goal is to enhance my overall muscle mass.	Great! Transitioning from machines to free weights is a fantastic step for building muscle mass. Here's a sample beginner resistance training plan you can adapt to enhance your overall muscle mass:<br>**Frequency**: 3 non-consecutive days per week (e.g., Monday, Wednesday, Friday)<br>**Warm-up**: 5–10 minutes of light cardio (jumping jacks, jogging) and dynamic stretches (arm circles, leg swings)<br><br>**Workout**:

Day 1: Lower Body & Core

Exercise	Sets	Repetition	Notes
Squats	3	8–12	Start with bodyweight or dumbbells, progress to barbel
Lunges	3	10–15	Dumbbells or bodyweight
Romanian Deadlifts	3	8–12	Dumbbells or bodyweight
Calf Raises	3	15–20	Bodyweight or with weight
Plank	3	30–60 s	–

Day 2: Upper Body & Core

Exercise	Sets	Repetition	Notes
Push-ups	3	as many reps as possible	Modify on knees if needed
Rows (seated cable, dumbbell, or barbell)	3	8–12	–
Overhead Press (dumbbells or barbell)	3	8–12	–
Bicep Curls	3	10–15	Dumbbells or cables
Tricep Extension	3	10–15	Dumbbells or cables
Anti-rotation press	3	10–15 per side	Cable or band

Day 3: Full Body & Core

Exercise	Sets	Repetition	Notes
Jump squats	3	10–15	–
Burpees	3	As many reps as possible	Modify if needed
Single-arm Dumbbell Rows	3	10–12 per side	–
Medicine Ball Slams	3	10–15	–
Dips	3	As many reps as possible	Modify if needed

Cool-down: 5–10 minutes of static stretches (e.g., hamstring stretch, chest stretch)

Progression:
- Gradually increase weight, reps, or sets as you get stronger.
- Aim to progress at least once every 2–3 weeks.
- If an exercise feels too easy, add weight or challenge yourself with a variation.

Important notes:
- This is a sample plan, and you may need to adjust it based on your fitness level and preferences.
- Always prioritize proper form over lifting heavy weights.
- Listen to your body and take rest days when needed.
- Consider consulting a certified personal trainer for personalized guidance and exercise modifications.

Remember, consistency, proper form, and progressive overload are key to building muscle mass. Good luck on your fitness journey!

S-TABLE 2. Conversation with Google Gemini and little information input (second attempt) on February 15, 2024

Inserted Question by Human	Response Generated by Gemini
**Please provide me with a resistance training plan to increase muscle hypertrophy.**	Unfortunately, I cannot create a personalized resistance training plan for you without more information. Providing a safe and effective plan requires understanding your individual fitness level, experience, goals, and access to equipment. However, I can offer some general guidelines and resources to help you design your own plan:<br><br>**General Guidelines:**<br>• **Frequency**: Aim for 2–4 resistance training sessions per week, allowing at least 48 hours of rest between sessions for the same muscle group.<br>• **Sets and reps:** For hypertrophy, focus on 3–6 sets of 8–12 repetitions per exercise.<br>• **Intensity**: Choose a weight that challenges you for the desired number of repetitions, ideally reaching fatigue in the last rep or two of each set. Aim for 70–85% of your 1-rep max (1RM).<br>• **Progression**: Gradually increase weight, sets, or reps over time to keep challenging your muscles and stimulate growth.<br>• **Compound exercises**: Prioritize compound exercises that work multiple muscle groups simultaneously, such as squats, deadlifts, lunges, presses, rows, and pull-ups.<br>• **Progressive overload**: This principle is crucial for muscle growth. It means gradually increasing the difficulty of your workouts over time, either by increasing weight, sets, reps, or exercise complexity.<br>• **Warm-up and cool-down**: Always perform a proper warm-up before each workout and a cool-down afterward to minimize injury risk.<br><br>**Resources:**<br>• National Strength and Conditioning Association (NSCA): https://www.nsca.com/<br>• American College of Sports Medicine (ACSM): https://www.acsm.org/<br>• Bodybuilding.com: https://www.bodybuilding.com/<br>• StrongLifts 5 × 5: https://stronglifts.com/stronglifts-5x5/<br><br>**Important Note:**<br>Before starting any new exercise program, please consult with a healthcare professional, especially if you have any underlying health conditions or injuries.<br><br>Remember, consistency is key. Stick to your program, track your progress, and adjust as needed to reach your goals!

S-TABLE 3. Conversation with Microsoft Copilot and little information input (first attempt) on February 15, 2024

Inserted Question by Human	Response Generated by Copilot
**Please provide me with a resistance training plan to increase muscle hypertrophy.**	Resistance training is a type of exercise that challenges your muscles by applying external forces, such as weights, bands, or your own bodyweight[1]. Resistance training can stimulate muscle growth, also known as hypertrophy, by increasing the size and number of your muscle fibers[2].<br><br>There are many factors that influence how effective your resistance training plan is for muscle hypertrophy, such as:<br>• The choice of exercises: You should include both compound and isolation exercises that target all the major muscle groups in your body. Compound exercises are those that involve multiple joints and muscles, such as squats, bench presses, and rows. Isolation exercises are those that focus on one joint and muscle, such as bicep curls, leg extensions, and lateral raises[2].<br>• The number of repetitions and sets: You should perform enough repetitions per set to fatigue your muscles, but not so many that you compromise your form or intensity. A common range for hypertrophy is 8 to 15 repetitions per set, but this can vary depending on your goals, experience, and preferences[2]. You should also do enough sets per exercise to stimulate muscle growth, but not so many that you overtrain or impair your recovery. A common range for hypertrophy is 3 to 6 sets per exercise, but this can also vary depending on the factors mentioned above[2].<br>• The frequency of training: You should train each muscle group at least twice a week, but not more than four times a week, to optimize muscle hypertrophy. This allows you to provide enough stimulus and recovery for your muscles to grow[2]. You can either follow a full-body routine, where you train all your muscles in one session, or a split routine, where you divide your muscles into different sessions, such as upper body and lower body, or push and pull[2].<br>• The intensity of training: You should train with a high level of effort and challenge your muscles to lift heavier weights over time. This is known as progressive overload, and it is the key to muscle hypertrophy. You can increase the intensity of your training by adding more weight, doing more repetitions or sets, reducing the rest time between sets, or using advanced techniques such as rest-pause, drop sets, or negatives[2].<br>• The rest time between sets: You should rest enough between sets to allow your muscles to recover and perform the next set with good form and intensity, but not so much that you lose the metabolic stress and tension that contribute to muscle hypertrophy. A common range for hypertrophy is 60 to 90 seconds, but this can vary depending on the exercise, the weight, and your goals[2].<br><br>Here is an example of a resistance training plan for muscle hypertrophy that you can follow for 8 weeks. It is based on a 4-day split routine, where you train your chest and triceps on Monday, your back and biceps on Tuesday, your legs on Thursday, and your shoulders and abs on Friday. You can adjust the exercises, weights, repetitions, sets, and rest times according to your own level and preferences.

****Monday: Chest and Triceps****

Exercise	Sets	Repetition	Rest
Bench Press	4	8	90 s
Incline Dumbbell Press	3	10–15	75 s
Cable Crossover	3	12–18	60 s
Triceps Pushdown	4	10–15	75 s
Overhead Triceps Extension	3	12–18	60 s

****Tuesday: Back and Biceps****

Exercise	Sets	Repetition	Rest
Lat Pulldown	4	8–12	90 s
Bent-Over Row	3	10–15	75 s
Seated Cable Row	3	12–18	60 s
Bicep Curl	4	10–15	75 s
Hammer Curl	3	12–18	60 s

S-TABLE 3. Continue.

Inserted Question by Human	Response Generated by Copilot

Thursday: Legs

Exercise	Sets	Repetition	Rest
Squat	4	8–12	90 s
Leg Press	3	10–15	75 s
Leg Extension	3	12–18	60 s
Leg Curl	4	10–15	75 s
Calf Raise	3	15–20	60 s

Friday: Shoulders and Abs

Exercise	Sets	Repetition	Rest
Shoulder Press	4	8–12	90 s
Lateral Raise	3	10–15	75 s
Front Raise	3	12–18	60 s
Rear Delt Fly	3	12–18	60 s
Crunch	3	15–20	60 s
Plank	3	30–60	60 s

I hope this helps you with your muscle hypertrophy goals. Remember to warm up before each session, cool down after each session, and stretch your muscles regularly. You should also eat a balanced diet that provides enough protein, carbohydrates, fats, and micronutrients to support your muscle growth and recovery. And don't forget to drink plenty of water and get enough sleep. Good luck and have fun! 😊

[1]: [The Hypertrophy Training Guide: How to Lift for Muscle Size](^2^)
[2]: [8 Week Mass Building Hypertrophy Workout | Muscle & Strength](^1^)

Resources:
(1) 8 Week Mass Building Hypertrophy Workout | Muscle & Strength. https://www.muscleandstrength.com/workouts/8-week-hypertrophy-workout.
(2) The Hypertrophy Training Guide: How to Lift for Muscle Size. https://outlift.com/hypertrophy-training-guide/.
(3) The Hypertrophy Training Guide: How to Lift for Muscle Size. https://outlift.com/hypertrophy-training-guide/.
(4) 8 Week Mass Building Hypertrophy Workout | Muscle & Strength. https://www.muscleandstrength.com/workouts/8-week-hypertrophy-workout.
(5) Optimizing Resistance Training Technique to Maximize Muscle Hypertrophy .... https://www.mdpi.com/2411-5142/9/1/9.

S-TABLE 4. Conversation with Microsoft Copilot and little information input (second attempt) on February 15, 2024

Inserted Question by Human	Response Generated by Copilot
**Please provide me with a resistance training plan to increase muscle hypertrophy.**	I'm sorry, but I cannot provide you with a personalized resistance training plan. That would require a thorough assessment of your goals, preferences, current level, medical history, and other factors. Only a qualified professional can design a suitable plan for you.<br><br>However, I can give you some general guidelines and examples of resistance training for muscle hypertrophy, based on the web search results that I found. Please note that these are not specific recommendations and you should consult with a professional before starting any new exercise or nutrition program.<br><br>According to the web search results, resistance training for muscle hypertrophy involves:<br>• Lifting weights that challenge your muscles and cause them to grow bigger and stronger. This can be done with free weights, machines, or bodyweight exercises[12].<br>• Increasing the weight, reps, sets, or frequency of your training over time to create progressive overload. This means that you are constantly challenging your muscles to adapt and improve[12].<br>• Doing a moderate number of reps (6–12) per set, with a moderate to high number of sets (3–6) per exercise, and a short to moderate rest period (30–90 seconds) between sets. This creates a balance between mechanical tension, metabolic stress, and muscle damage, which are the main factors that stimulate muscle growth[123].<br>• Training each muscle group at least twice a week, with a total weekly volume of 10–20 sets per muscle group. This ensures that you are stimulating your muscles frequently and sufficiently enough to grow[12].<br>• Using a variety of exercises that target different parts of your muscles, such as compound lifts (e.g., squats, bench press, rows) and isolation lifts (e.g., leg extensions, chest flyes, biceps curls). This helps you develop a balanced and symmetrical physique[12].<br>• Incorporating advanced training techniques, such as drop sets, rest-pause sets, or negatives, to increase the intensity and challenge of your workouts. These techniques involve reducing the weight, resting briefly, or slowing down the lowering phase of the lift after reaching failure, and then continuing to perform more reps. This can help you squeeze out more growth from your muscles[13].<br><br>Here is an example of a four-day split resistance training plan for muscle hypertrophy, based on one of the web search results[1]:

Day 1: Chest, Shoulders, and Triceps

Exercise	Sets	Repetition	Rest
Bench Press	4	6–8	3 min.
Incline Dumbbell Press	3	8–10	2 min.
Chest Flyes	3	12–15	90 s
Lateral Raises	3	12–15	90 s
Overhead Press	3	8–10	2 min.
Triceps Extension	3	12–15	90 s
Close-Grip Bench Press	3	8–10	2 min.

Day 2: Back, Biceps, and Core

Exercise	Sets	Repetition	Rest
Deadlift	4	6–8	3 min.
Rows	4	8–10	2 min.
Pulldown	3	10–12	2 min.
Pullover	3	12–15	90 s
Biceps Curls	4	12–15	90 s
Hammer Curls	3	10–12	2 min.
Weighted Sit-Ups	3	15–20	90 s
Plank	3	30–90	90 s

Day 3: Rest

S-TABLE 4. Continue.

Inserted Question by Human	Response Generated by Copilot

Day 4: Legs

Exercise	Sets	Repetition	Rest
Squats	4	6–8	3 min.
Leg Press	4	10–12	2 min.
Leg Extensions	3	15–20	90 s
Leg Curls	3	12–15	90 s
Calf Raises	4	15–20	90 s
Lunges	3	10–12	2 min.

Day 5: Rest

Day 6: Repeat Day 1

Day 7: Repeat Day 2

This is just one example of a possible resistance training plan for muscle hypertrophy. You can modify it according to your own needs, preferences, and goals. You can also find more examples and information on the web search results that I found.

I hope this helps you get an idea of how to train for muscle hypertrophy. If you have any questions or feedback, please let me know. 😊

Resources:

(1) 8 Week Mass Building Hypertrophy Workout | Muscle & Strength. https://www.muscleandstrength.com/workouts/8-week-hypertrophy-workout.
(2) The Hypertrophy Training Guide: How to Lift for Muscle Size. https://outlift.com/hypertrophy-training-guide/.
(3) These Researchers Reveal the Right Way to Train for More Muscle Mass. https://barbend.com/training-for-muscle-mass-according-to-researchers/.

S-TABLE 5. Conversation with Google Gemini and detailed information input (first attempt) on February 15, 2024

Inserted Question by Human:

Please provide me with a resistance training plan to increase muscle hypertrophy over the next 16 weeks. I am a 25-year-old male and have been doing resistance training 4 times a week for the past 8 years. Previous resistance training sessions have lasted 90 minutes. I have access to free weights and machines, both of which I would like to use. I also have training equipment such as belts, straps, bandages that I can use, and I have a body composition scale for monitoring purposes. My body weight is 80 kg with 12% body fat. I am 180 cm tall. I would like to increase the frequency to 5–6 times a week. I want to increase my total muscle mass as much as possible, although I am at an advanced level. I want to emphasise my arms as they are proportionally smaller than the rest of my body. I like to train with 3 seconds long eccentric actions, while the concentric action is explosive. My one-rep maximums in the squat, bench press and deadlift are 200 kg, 140 kg and 230 kg respectively. Overall, I want to incorporate advanced training strategies such as drop sets because I enjoy them. I also want to focus on my nutrition and recovery for muscle hypertrophy.

To give you an idea of how I used to train, here is my previous resistance training programme:

Day 1 & Day 3: Leg day & core

Exercises	Sets	repetitions	intensity	Rest periods
Squats	4	8	3 reps in reserve	3 min.
Leg Press	3	10	3 reps in reserve	90 s
Leg ext.	3	15–20	1–2 reps in reserve	90 s
Calf raises	3	15	1–2 reps in reserve	90 s
Weighted sit ups	2	15–20	1–2 reps in reserve	90 s

Day 2 & Day 4: Upper body

Exercises	Sets	repetitions	intensity	Rest periods
Bench press	4	8	3 reps in reserve	3 min.
Rows	4	8	3 reps in reserve	3 min.
Incline bench press	3	10	3 reps in reserve	3 min.
Pulldown	3	12	3 reps in reserve	3 min.
Lateral raises	2	15–20	1–2 reps in reserve	90 s
Biceps curls	3	15	1–2 reps in reserve	90 s
Triceps ext.	3	15	1–2 reps in reserve	90 s

Sample Weekly Structure:

Day	Focus	Exercises	Sets	Reps	Rest	Notes
Mon.	Push	Chest & Triceps	4–5	6–8	2–3 min	Explosive concentric, 3 s eccentric
Tues.	Pull	Back & Biceps	4–5	8–10	2–3	Explosive concentric, 3 s eccentric
Wed.	Legs	Quads & Hamstrings	4–5	8–12	2–3	Explosive concentric, 3 s eccentric
Thurs.	Rest	–	–	–	–	–
Fri.	Push	Shoulders & Triceps	4–5	8–12	2–3	Include lateral raises with drop sets
Sat.	Pull	Back & Biceps	4–5	10–12	2–3	Include bicep curls with drop sets
Sun.	Legs	Glutes & Calves	4–5	12–15	2–3	Include calf raises with drop sets

Response Generated by Gemini:

Personalized Resistance Training Plan for Muscle Hypertrophy (16 Weeks)

Disclaimer: This plan is provided for informational purposes only and should not be considered a substitute for professional medical or fitness advice. Always consult with a healthcare professional before starting any new exercise program, especially if you have any underlying health conditions.

Based on your information, here's a sample 16-week resistance training plan:

- Frequency: 5 days per week (with 2 rest days)
- Split: Push-Pull-Legs (PPL) with an emphasis on arms
- Training Duration: 60–75 minutes per session
- Warm-up: 10–15 minutes of dynamic stretches and light cardio.
- Cool-down: 5–10 minutes of static stretches and foam rolling.
- Exercise Selection: You've requested a balance between free weights and machines, so the plan incorporates both.
- Advanced Techniques: Drop sets will be included in various exercises for further muscle stimulation.
- Nutrition and Recovery: Tips for optimizing these aspects are provided at the end.

S-TABLE 5. Continue.

Specific Exercise Examples:
- Push:
  - Barbell Bench Press
  - Dumbbell Incline Press
  - Machine Shoulder Press
  - Overhead Tricep Extensions
  - Dips
- Pull:
  - Bent-Over Barbell Row
  - Seated Cable Row
  - Pull-Ups (assisted if needed)
  - Barbell Bicep Curls
  - Hammer Curls
- Legs:
  - Barbell Squats
  - Leg Press
  - Romanian Deadlifts
  - Leg Extensions
  - Hamstring Curls
  - Glute Bridges
  - Calf Raises

Progression:
Increase weight by 2.5–5 kg once you can comfortably complete the prescribed reps and sets.
Use different rep ranges throughout the program (e.g., Week 1–4 = 8 reps, Week 5–8 = 10 reps, Week 9–12 = 12 reps).
Gradually increase training volume (sets and reps) over time, but ensure adequate recovery.

Nutrition and Recovery:
Consume 1.5–2 grams of protein per kilogram of body weight daily.
Focus on whole foods like fruits, vegetables, lean protein, and complex carbohydrates.
Stay hydrated by drinking plenty of water throughout the day.
Get 7–8 hours of quality sleep each night.
Consider using foam rolling and other recovery modalities to reduce muscle soreness.

Remember:
- Adjust the plan based on your individual needs and progress.
- Pay close attention to proper form and technique.
- Listen to your body and take rest days when needed.
- Seek professional guidance if you have any concerns or injuries.

This is just a sample plan, and you may need to adjust it based on your personal preferences and progress. Remember, consistency and proper form are key to achieving your muscle hypertrophy goals.

Resource:
ideasocity.com/what-are-the-health-benefits-of-a-morning-wal**k**/"

S-TABLE 6. Conversation with Google Gemini and detailed information input (second attempt) on February 15, 2024

Inserted Question by Human:
Please provide me with a resistance training plan to increase muscle hypertrophy over the next 16 weeks. I am a 25-year-old male and have been doing resistance training 4 times a week for the past 8 years. Previous resistance training sessions have lasted 90 minutes. I have access to free weights and machines, both of which I would like to use. I also have training equipment such as belts, straps, bandages that I can use, and I have a body composition scale for monitoring purposes. My body weight is 80 kg with 12% body fat. I am 180 cm tall. I would like to increase the frequency to 5–6 times a week. I want to increase my total muscle mass as much as possible, although I am at an advanced level. I want to emphasise my arms as they are proportionally smaller than the rest of my body. I like to train with 3 seconds long eccentric actions, while the concentric action is explosive. My one-rep maximums in the squat, bench press and deadlift are 200 kg, 140 kg and 230 kg respectively. Overall, I want to incorporate advanced training strategies such as drop sets because I enjoy them. I also want to focus on my nutrition and recovery for muscle hypertrophy.

To give you an idea of how I used to train, here is my previous resistance training programme:

Day 1 & Day 3: Leg day & core

Exercises	Sets	repetitions	intensity	Rest periods
Squats	4	8	3 reps in reserve	3 min.
Leg Press	3	10	3 reps in reserve	90 s
Leg ext.	3	15–20	1–2 reps in reserve	90 s
Calf raises	3	15	1–2 reps in reserve	90 s
Weighted sit ups	2	15–20	1–2 reps in reserve	90 s

Day 2 & Day 4: Upper body

Exercises	Sets	repetitions	intensity	Rest periods
Bench press	4	8	3 reps in reserve	3 min.
Rows	4	8	3 reps in reserve	3 min.
Incline bench press	3	10	3 reps in reserve	3 min.
Pulldown	3	12	3 reps in reserve	3 min.
Lateral raises	2	15–20	1–2 reps in reserve	90 s
Biceps curls	3	15	1–2 reps in reserve	90 s
Triceps ext.	3	15	1–2 reps in reserve	90 s

Response Generated by Gemini:
"Resistance Training Plan for Muscle Hypertrophy (16 Weeks)
Disclaimer: This is a general template and not a substitute for personalized guidance from a qualified trainer or coach. Consider consulting a professional before implementing this plan, especially with your advanced training experience and specific goals.

Overall Structure:
- Frequency: 5–6 times per week (consider rotating muscle groups to manage volume)
- Session duration: 60–90 minutes
- Rest between sets: 60–120 seconds (depending on exercise and rep range)
- Progression: Focus on progressive overload through increasing weight, sets, reps, or exercise difficulty

Split Options:

Push-Pull-Legs:
- Push (chest, shoulders, triceps)
- Pull (back, biceps)
- Legs (quads, hamstrings, calves)
- Repeat, adding an optional core day if desired.

Upper-Lower:
- Upper body (push and pull muscles)
- Lower body (quads, hamstrings, calves)
- Repeat, adding an optional core day if desired.

S-TABLE 6. Continue.

Bro-Split:
- Chest & triceps
- Back & biceps
- Legs
- Shoulders & calves
- Rest

Exercise Selection:
- Choose compound movements for major muscle groups (squats, deadlifts, rows, presses)
- Include isolation exercises to target specific areas (biceps curls, triceps extensions, lateral raises)
- Don't neglect core exercises for stability and injury prevention

Advanced Techniques:
- Drop sets, supersets, pyramid sets, negatives
- Focus on mind-muscle connection and controlled movements
- Utilize training equipment strategically (belts, straps, etc.)

Sample Week (Push-Pull-Legs Split):

Day 1: Push

Exercise	Sets	Repetition
Barbell Bench Press	3	8–12
Incline Dumbbell Press	3	10–12
Overhead Press	3	8–10
Push–ups	3	failure
Triceps Pushdowns	3	12–15

Day 2: Pull

Exercise	Sets	Repetition
Barbell Deadlift	3	5–8
Pull–ups	3	failure, assisted if needed
Barbell Rows	3	8–12
Seated Cable Rows	3	12–15
Bicep Curls	3	10–12

Day 3: Legs

Exercise	Sets	Repetition
Squats	3	8–12
Leg Press	3	10–12
Romanian Deadlift	3	12–15
Calf Raises	3	15–20
Hanging Leg Raises	3	failure

Day 4: Push (Variation)

Exercise	Sets	Repetition
Incline Barbell Press	3	8–12
Arnold Press	3	10–12
Dips	3	failure
Overhead Triceps Extension	3	12–15

Day 5: Pull (Variation)

Exercise	Sets	Repetition
Barbell Bent-Over Rows	3	8–12
Lat Pulldowns	3	10–12
Face Pulls	3	12–15
Hammer Curls	3	10–12

Day 6: Legs (Variation)

Exercise	Sets	Repetition
Bulgarian Split Squats	3	10–12 per leg
Leg Extension	3	12–15
Hamstring Curls	3	12–15
Walking Lunges	3	15–20 reps per leg
Plank	3	failure

Tips:
- Track your progress: Monitor lifts, body composition, and workout performance.
- Prioritize sleep and recovery: Aim for 7–8 hours of sleep per night and utilize active recovery methods.
- Fuel your body: Focus on a balanced diet with sufficient protein (0.8–1.2 g per kg body weight) and carbohydrates.
- Listen to your body: Take rest days when needed and adjust the plan as necessary.
- Remember: This is just a starting point. You may need to adjust the plan based on your individual needs and preferences.

S-TABLE 7. Conversation with Microsoft Copilot and detailed information input (first attempt) on February 15, 2024

Inserted Question by Human:

Please provide me with a resistance training plan to increase muscle hypertrophy over the next 16 weeks. I am a 25-year-old male and have been doing resistance training 4 times a week for the past 8 years. Previous resistance training sessions have lasted 90 minutes. I have access to free weights and machines, both of which I would like to use. I also have training equipment such as belts, straps, bandages that I can use, and I have a body composition scale for monitoring purposes. My body weight is 80 kg with 12% body fat. I am 180 cm tall. I would like to increase the frequency to 5–6 times a week. I want to increase my total muscle mass as much as possible, although I am at an advanced level. I want to emphasise my arms as they are proportionally smaller than the rest of my body. I like to train with 3 seconds long eccentric actions, while the concentric action is explosive. My one-rep maximums in the squat, bench press and deadlift are 200 kg, 140 kg and 230 kg respectively. Overall, I want to incorporate advanced training strategies such as drop sets because I enjoy them. I also want to focus on my nutrition and recovery for muscle hypertrophy.

To give you an idea of how I used to train, here is my previous resistance training programme:

Day 1 & Day 3: Leg day & core

Exercises	Sets	repetitions	intensity	Rest periods
Squats	4	8	3 reps in reserve	3 min.
Leg Press	3	10	3 reps in reserve	90 s
Leg ext.	3	15–20	1–2 reps in reserve	90 s
Calf raises	3	15	1–2 reps in reserve	90 s
Weighted sit ups	2	15–20	1–2 reps in reserve	90 s

Day 2 & Day 4: Upper body

Exercises	Sets	repetitions	intensity	Rest periods
Bench press	4	8	3 reps in reserve	3 min.
Rows	4	8	3 reps in reserve	3 min.
Incline bench press	3	10	3 reps in reserve	3 min.
Pulldown	3	12	3 reps in reserve	3 min.
Lateral raises	2	15–20	1–2 reps in reserve	90 s
Biceps curls	3	15	1–2 reps in reserve	90 s
Triceps ext.	3	15	1–2 reps in reserve	90 s

Response Generated by Copilot:

Hello, this is Copilot. I'm glad you are interested in improving your muscle hypertrophy. Based on your goals, preferences, and current level, I have created a customized resistance training plan for you. This plan is designed to increase your training volume, intensity, and frequency, while also incorporating advanced techniques such as drop sets, rest-pause sets, and negatives. It also includes some tips on nutrition and recovery to optimize your muscle growth.

Here is your new resistance training plan for the next 16 weeks:

Day 1 & Day 5: Chest, Shoulders & Triceps

Exercise	Sets	Repetition	Intensity	Rest periods
Bench press	4	6–8	2 reps in reserve	3 min.
Incline bench press	3	8–10	2 reps in reserve	2 min.
Dumbbell flys	3	12–15	1 rep in reserve	90 s
Drop set: Cable crossover	3	15–20	failure	90 s
Overhead press	4	6–8	2 reps in reserve	3 min.
Lateral raises	3	12–15	1 rep in reserve	90 s
Drop set: Front raises	2	15–20	failure	90 s
Skullcrushers	3	8–10	2 reps in reserve	2 min.
Triceps pushdown	3	12–15	1 rep in reserve	90 s
Drop set: Overhead triceps extension	2	15–20	failure	90 s

S-TABLE 7. Continue.

Day 2 & Day 6: Back, Biceps & Abs

Exercise	Sets	Repetition	Intensity	Rest periods
Deadlift	4	6–8	2 reps in reserve	3 min.
Barbell row	3	8–10	2 reps in reserve	2 min.
Pulldown	3	12–15	1 rep in reserve	90 s
Drop set: Seated cable row	2	15–20	failure	90 s
Barbell curl	3	8–10	2 reps in reserve	2 min.
Hammer curl	3	12–15	1 rep in reserve	90 s
Drop set: Preacher curl	2	15–20	failure	90 s
Weighted crunch	3	15–20	1 rep in reserve	90 s
Hanging leg raise	3	15–20	1 rep in reserve	90 s
Drop set: Plank	3	60 s	failure	90 s

Day 3 & Day 7: Legs & Calves

Exercise	Sets	Repetition	Intensity	Rest periods
Squat	4	6–8	2 reps in reserve	3 min.
Leg press	3	8–10	2 reps in reserve	2 min.
Rest–pause set: Leg extension	3	15–20	failure	90 s
Romanian deadlift	3	8–10	2 reps in reserve	2 min.
Rest–pause set: Leg curl	3	15–20	failure	90 s
Standing calf raise	4	15–20	1 rep in reserve	90 s
Seated calf raise	4	15–20	1 rep in reserve	90 s
Negative: Donkey calf raise	2	10–15	failure	90 s

Day 4 & Day 8: Rest

Some notes on the plan:

- You will train each muscle group twice a week, with at least 48 hours of rest between sessions. This will allow you to stimulate more muscle protein synthesis and recovery.
- You will use a moderate rep range of 6–15 reps, which is optimal for muscle hypertrophy. You will also vary the rep ranges within each workout to target different muscle fibers and metabolic pathways.
- You will train with a high intensity of 1–2 reps in reserve, meaning you will stop the set when you have 1 or 2 reps left in the tank. This will ensure you are challenging your muscles enough to induce growth, without risking overtraining or injury.
- You will use advanced techniques such as drop sets, rest-pause sets, and negatives to increase the metabolic stress and muscle damage, which are key factors for hypertrophy. A drop set is when you reduce the weight by 20–30% after reaching failure and continue the set until failure again. A rest-pause set is when you rest for 10–15 seconds after reaching failure and resume the set until failure again. A negative is when you lower the weight slowly and under control for 3–5 seconds, focusing on the eccentric phase of the movement.
- You will rest for 90 seconds to 3 minutes between sets, depending on the exercise and the intensity. This will allow you to recover enough to perform the next set with good form and intensity, while also keeping the workout duration reasonable.
- You will perform the exercises with a 3-second eccentric and an explosive concentric, as you prefer. This will increase the time under tension and the muscle activation, while also improving your power and strength.
- You will emphasize your arms by performing more sets and exercises for them, as well as using drop sets for each arm exercise. This will create more stimulus and pump for your arms, which are lagging behind the rest of your body.

S-TABLE 7. Continue.

Some tips on nutrition and recovery:

- To maximize your muscle hypertrophy, you will need to eat in a caloric surplus of around 300–500 calories above your maintenance level. This will provide enough energy and nutrients for your muscles to grow and repair. You can use a [calorie calculator] to estimate your maintenance level and adjust it according to your progress and goals.
- You will also need to consume enough protein, which is the main building block of muscle tissue. Aim for around 1.6–2.2 grams of protein per kilogram of body weight per day, which is around 128–176 grams for you. You can get protein from animal sources such as meat, eggs, dairy, and fish, or plant sources such as soy, beans, nuts, and seeds. You can also supplement with whey or casein protein if you struggle to meet your protein needs from food alone.
- You will also benefit from consuming enough carbohydrates, which are the main fuel source for your muscles and brain. Carbohydrates will replenish your muscle glycogen, which is the stored form of glucose that powers your workouts. They will also spike your insulin, which is a hormone that helps shuttle nutrients into your muscle cells and prevents muscle breakdown. Aim for around 4–6 grams of carbohydrates per kilogram of body weight per day, which is around 320–480 grams for you. You can get carbohydrates from whole grains, fruits, vegetables, legumes, and starchy foods such as rice, potatoes, and pasta.
- You will also need to consume enough healthy fats, which are essential for your hormone production, cell membrane function, and overall health. Fats will also help you feel fuller and more satisfied, which can prevent overeating and cravings. Aim for around 0.8–1.2 grams of fat per kilogram of body weight per day, which is around 64–96 grams for you. You can get fats from oils, nuts, seeds, avocados, olives, and fatty fish such as salmon, mackerel, and sardines.
- You will also need to drink enough water, which is vital for your hydration, metabolism, digestion, and performance. Water will also help you flush out toxins and waste products from your body, as well as regulate your body temperature and blood pressure. Aim for around 3–4 liters of water per day, or more if you sweat a lot or live in a hot climate. You can also drink other fluids such as tea, coffee, juice, and milk, but avoid sugary drinks, alcohol, and excessive caffeine, as they can dehydrate you and interfere with your sleep and recovery.
- Speaking of sleep and recovery, you will also need to prioritize them, as they are crucial for your muscle growth and overall health. Sleep is when your body repairs and grows your muscles, as well as restores your nervous system and hormonal balance. Aim for around 7–9 hours of quality sleep per night, and try to keep a consistent sleep schedule. You can also take naps during the day if you feel tired or need to catch up on some sleep. To improve your sleep quality, you can avoid caffeine, alcohol, and screens before bed, as well as create a dark, quiet, and comfortable sleeping environment.
- Recovery is also important, as it allows your muscles and joints to heal and adapt to the training stimulus. You can enhance your recovery by doing some light cardio, stretching, foam rolling, massage, or other forms of active recovery on your rest days or after your workouts. You can also use some recovery tools such as belts, straps, bandages, and ice packs to support your muscles and joints and prevent injuries. You can also monitor your recovery by using a body composition scale, as well as tracking your weight, body fat percentage, muscle mass, strength, performance, mood, and energy levels.

I hope this plan helps you achieve your muscle hypertrophy goals. Please let me know if you have any questions or feedback. I’m always happy to help. 😊

S-TABLE 8. Conversation with Microsoft Copilot and detailed information input (second attempt) on February 15, 2024

Inserted Question by Human:

Please provide me with a resistance training plan to increase muscle hypertrophy over the next 16 weeks. I am a 25-year-old male and have been doing resistance training 4 times a week for the past 8 years. Previous resistance training sessions have lasted 90 minutes. I have access to free weights and machines, both of which I would like to use. I also have training equipment such as belts, straps, bandages that I can use, and I have a body composition scale for monitoring purposes. My body weight is 80 kg with 12% body fat. I am 180 cm tall. I would like to increase the frequency to 5–6 times a week. I want to increase my total muscle mass as much as possible, although I am at an advanced level. I want to emphasise my arms as they are proportionally smaller than the rest of my body. I like to train with 3 seconds long eccentric actions, while the concentric action is explosive. My one-rep maximums in the squat, bench press and deadlift are 200 kg, 140 kg and 230 kg respectively. Overall, I want to incorporate advanced training strategies such as drop sets because I enjoy them. I also want to focus on my nutrition and recovery for muscle hypertrophy.

To give you an idea of how I used to train, here is my previous resistance training programme:

Day 1 & Day 3: Leg day & core

Exercises	Sets	repetitions	intensity	Rest periods
Squats	4	8	3 reps in reserve	3 min.
Leg Press	3	10	3 reps in reserve	90 s
Leg ext.	3	15–20	1–2 reps in reserve	90 s
Calf raises	3	15	1–2 reps in reserve	90 s
Weighted sit ups	2	15–20	1–2 reps in reserve	90 s

Day 2 & Day 4: Upper body

Exercises	Sets	repetitions	intensity	Rest periods
Bench press	4	8	3 reps in reserve	3 min.
Rows	4	8	3 reps in reserve	3 min.
Incline bench press	3	10	3 reps in reserve	3 min.
Pulldown	3	12	3 reps in reserve	3 min.
Lateral raises	2	15–20	1–2 reps in reserve	90 s
Biceps curls	3	15	1–2 reps in reserve	90 s
Triceps ext.	3	15	1–2 reps in reserve	90 s

Response Generated by Copilot:

Hello, this is Copilot. I'm glad you are interested in improving your muscle hypertrophy. Based on your goals, experience, and preferences, I have created a resistance training plan for you that will last for 16 weeks. This plan is divided into four phases, each lasting four weeks. Each phase has a different focus and intensity, and you will train five times a week, alternating between upper and lower body sessions. Here is an overview of the plan:

Phase 1: Accumulation

- The goal of this phase is to increase your training volume and stimulate muscle growth by using moderate weights and high reps.
- You will perform 3–4 sets of 12–15 reps per exercise, with 60–90 seconds of rest between sets.
- You will use a variety of exercises, including free weights, machines, and cables, to target all the major muscle groups.
- You will also use some advanced techniques, such as drop sets and rest-pause sets, to increase the intensity and challenge your muscles.
- You will train your arms twice a week, as they are your priority area.

Phase 2: Intensification

- The goal of this phase is to increase your training intensity and strength by using heavier weights and lower reps.
- You will perform 4–5 sets of 6–10 reps per exercise, with 2–3 minutes of rest between sets.
- You will focus on compound exercises, such as squats, bench press, and deadlifts, to maximize your muscle recruitment and overload.
- You will also use some advanced techniques, such as negatives and forced reps, to push your muscles beyond failure and induce more damage and growth.
- You will train your arms once a week, but with higher intensity and volume.

Phase 3: Realization

- The goal of this phase is to realize your strength and size gains by using near-maximal weights and low reps.
- You will perform 5–6 sets of 3–5 reps per exercise, with 3–5 minutes of rest between sets.
- You will focus on the main lifts, such as squats, bench press, and deadlifts, and try to increase your one-rep maxes.
- You will also use some advanced techniques, such as clusters and singles, to improve your neural efficiency and power output.
- You will train your arms once a week, but with lower intensity and volume.

S-TABLE 8. Continue.

Phase 4: Deload

- The goal of this phase is to reduce your training volume and intensity and allow your muscles and nervous system to recover and adapt.
- You will perform 2–3 sets of 8–12 reps per exercise, with 60–90 seconds of rest between sets.
- You will use a variety of exercises, including free weights, machines, and cables, to maintain your muscle mass and activation.
- You will also use some basic techniques, such as tempo and pauses, to improve your form and technique.
- You will train your arms once a week, but with minimal intensity and volume.

Here is an example of how your weekly schedule might look like:

- Monday: Upper body
- Tuesday: Lower body
- Wednesday: Rest
- Thursday: Upper body
- Friday: Lower body
- Saturday: Rest
- Sunday: Rest

Here is an example of how your first week of phase 1 might look like:

Day 1: Upper body

Exercise	Sets	Repetition	Rest periods	Notes
Bench press	3	15	90 s	Rest-pause on the last set
Incline dumbbell press	3	15	90 s	Drop set on the last set
Cable crossover	3	15	60 s	–
Barbell row	3	15	90 s	Rest-pause on the last set
Barbell row	3	15	90 s	Drop set on the last set
Face pull	3	15	60 s	–
Biceps curl	4	15	60 s	Drop set on the last set
Triceps pushdown	4	15	60 s	Drop set on the last set

Day 2: Lower body

Exercise	Sets	Repetition	Rest periods	Notes
Squat	3	15	90 s	Rest-pause on the last set
Leg press	3	15	90 s	Drop set on the last set
Leg curl	3	15	60 s	–
Leg extension	3	15	60 s	–
Calf raise	4	15	60 s	Drop set on the last set
Weighted sit-ups	3	15	60 s	–
Plank	3	60 s	60 s	–

Day 3: Rest

S-TABLE 8. Continue.

Day 4: Upper body

Exercise	Sets	Repetition	Rest periods	Notes
Military press	3	15	90 s	Rest–pause on the last set
Dumbbell lateral raise	3	15	90 s	Drop set on the last set
Cable rear delt fly	3	15	60 s	–
Close–grip bench press	3	15	90 s	Rest–pause on the last set
Dumbbell skullcrusher	3	15	90 s	Drop set on the last set
Cable triceps extension	3	15	60 s	–
Chin–up	3	15	90 s	Rest–pause on the last set
Dumbbell curl	3	15	90 s	Drop set on the last set
Cable curl	3	15	60 s	–

Day 5: Lower body

Day 6: Rest

Day 7: Rest

This is just an example of how your plan might look like. You can adjust the exercises, sets, reps, and rest periods according to your preferences and availability of equipment. However, make sure to follow the main principles and guidelines of each phase and the overall plan.
As for your nutrition and recovery, here are some general tips to help you optimize your muscle hypertrophy:
- Eat enough calories to support your training and growth. A good starting point is to multiply your body weight in kilograms by 40–45. This will give you your daily calorie intake in calories. For example, if you weigh 80 kg, you should aim for 3200–3600 calories per day.
- Eat enough protein to stimulate muscle protein synthesis and repair. A good target is to consume 1.6–2.2 grams of protein per kilogram of body weight per day. For example, if you weigh 80 kg, you should aim for 128–176 grams of protein per day.
- Eat enough carbohydrates to fuel your workouts and replenish your glycogen stores. A good range is to consume 3–5 grams of carbohydrates per kilogram of body weight per day. For example, if you weigh 80 kg, you should aim for 240–400 grams of carbohydrates per day.
- Eat enough fats to support your hormonal health and cell function. A good minimum is to consume 0.6–1 gram of fat per kilogram of body weight per day. For example, if you weigh 80 kg, you should aim for 48–80 grams of fat per day.
- Eat a variety of foods, including fruits, vegetables, whole grains, lean meats, dairy, nuts, seeds, and healthy oils. This will help you get all the micronutrients and phytochemicals that are essential for your health and performance.
- Drink enough water to stay hydrated and prevent dehydration. A good rule of thumb is to drink at least 0.03 liters of water per kilogram of body weight per day. For example, if you weigh 80 kg, you should drink at least 2.4 liters of water per day.
- Supplement wisely and only if needed. Some supplements that may help you with your muscle hypertrophy are creatine, whey protein, caffeine, beta-alanine, and citrulline malate. However, these are not mandatory and should not replace a balanced diet.
- Sleep enough to recover and grow. A good goal is to get at least 7–9 hours of quality sleep per night. This will help you reduce stress, improve your hormone levels, and enhance your muscle growth and repair.
- Manage your stress and avoid overtraining. Stress can impair your recovery and performance, as well as increase your risk of injury and illness. Some ways to manage your stress are to meditate, breathe deeply, listen to music, read a book, or do something you enjoy. Also, avoid overtraining by following your plan, listening to your body, and taking deloads when needed.

I hope this helps

S-TABLE 9. Descriptive analysis (median and range) and results of the significance testing of remaining training plans generated by Google Gemini and Microsoft Copilot. Likert-Scale Ratings were from 1 (“bad”) to 5 (“good”) with 0 indicating “not applicable”.

		Median (Range)							
	**Relevant aspects when designing a training plan**	**GGL1**	**GGL2**	**GPT-4L1**	**GPT-4L2**	**GGD1**	**GGD2**	**GPT-4D1**	**GPT-4D2**
General Aspects	Overall training plan	2 (2)	0 (3)	3 (2)	3 (4)	3 (3)	3 (3)	4 (3)	3.5 (4)
	Health screening	2 (5)	2.5 (5)	0 (3)	2 (5)	1.5 (5)	0.5 (5)	0 (4)	0 (5)
	Defined goal	2 (4)	0.5 (5)	2 (4)	3.5 (5)	4 (5)	3 (5)	4 (5)	4 (5)
	Overall testing procedure	0 (4)	0 (2)	0 (2)	0 (3)	0 (4)	0 (4)	0 (5)	0 (5)
	Testing procedure regarding initial performance status	0 (4)	0 (2)	0 (5)	0 (4)	0 (5)	0 (5)	0 (5)	0 (5)
	Testing procedure regarding assessment of individual training variables	0 (4)	0 (2)	0 (4)	0 (4)	0 (4)	0 (4)	0 (5)	0 (5)
	Testing procedure regarding assessment of training effects	0 (4)	0 (2)	0 (5)	0 (3)	0 (4)	0.5 (4)	0 (5)	0 (4)
	Overall monitoring procedure	0 (3)	0 (2)	0 (4)	0 (3)	0 (4)	0.5 (4)	0.5 (5)	0 (5)
Summary Rating	**< 3**	**n = 8**	**n = 8**	**n = 7**	**n = 6**	**n = 6**	**n = 6**	**n = 6**	**n = 6**
	**3**	**n = 0**	**n = 0**	**n = 1**	**n = 1**	**n = 2**	**n = 2**	**n = 0**	**n = 0**
	**> 3**	**n = 0**	**n = 0**	**n = 0**	**n = 1**	**n = 0**	**n = 0**	**n = 2**	**n = 2**
Training principles	Principle of specificity	2 (4)	2 (4)	3 (5)	4 (5)	4 (4)	4 (4)	5 (4)	4 (3)
	Principle of progressive overload	2.5 (2)	3 (5)	4 (3)	3 (2)	4 (5)	4 (2)	3 (5)	4 (5)
	Principle of variation	2 (4)	0 (3)	3 (5)	3 (5)	3.5 (5)	4 (5)	4 (5)	4 (5)
	Principle of recovery	2.5 (4)	3 (5)	3 (3)	3 (5)	3.5 (4)	3.5 (3)	4 (4)	4 (3)
Summary Rating	**< 3**	**n = 4**	**n = 2**	**n = 0**	**n = 0**	**n = 0**	**n = 0**	**n = 0**	**n = 0**
	**3**	**n = 0**	**n = 2**	**n = 3**	**n = 3**	**n = 0**	**n = 0**	**n = 1**	**n = 0**
	**> 3**	**n = 0**	**n = 0**	**n = 1**	**n = 1**	**n = 4**	**n = 4**	**n = 3**	**n = 4**
Basic strength training aspects	Exercise selection	2 (3)	0 (4)	4 (3)	4 (4)	4 (5)	4 (3)	4 (3)	4 (3)
	Exercise order	3.5 (4)	0 (2)	4 (4)	4 (2)	3.5 (4)	4 (3)	4 (4)	4 (4)
	Weekly training frequency per muscle	2.5 (2)	2 (5)	3 (3)	3 (3)	4 (5)	4 (3)	4 (3)	4 (3)
	Training intensity per exercise	3 (5)	3 (4)	3 (3)	3 (5)	2 (4)	2 (5)	4 (3)	2.5 (5)
	Repetition range per exercise	3 (4)	3 (4)	3 (3)	4 (4)	2.5 (3)	3 (3)	4.5 (4)	4 (4)
	Overall training volume	3 (3)	0 (3)	3 (3)	4 (4)	3 (4)	4 (3)	3 (4)	4 (4)
	Number of sets per muscle per week	2.5 (3)	0 (5)	3 (2)	3 (3)	3 (4)	4 (3)	3.5 (4)	4 (3)
	Rest periods	2 (5)	0 (2)	3.5 (3)	4 (4)	3 (3)	3 (4)	4 (4)	4 (4)
	Exercise technique	1 (4)	0 (3)	0 (3)	0 (4)	0 (4)	0 (4)	0.5 (5)	1 (4)
Summary Rating	**< 3**	**n = 5**	**n = 7**	**n = 1**	**n = 1**	**n = 3**	**n = 2**	**n = 1**	**n = 2**
	**3**	**n = 3**	**n = 2**	**n = 5**	**n = 3**	**n = 3**	**n = 2**	**n = 1**	**n = 0**
	**> 3**	**n = 1**	**n = 0**	**n = 3**	**n = 5**	**n = 3**	**n = 5**	**n = 7**	**n = 7**
Advanced training aspects	Advanced exercise methods	0 (1)	0 (2)	0 (1)	0 (3)	3.5 (5)	0 (4)	3 (5)	0 (5)
	Time under tension	0 (2)	0 (2)	0 (3)	0 (3)	2.5 (5)	0 (4)	1 (5)	0 (5)
	Set end point	1 (5)	0 (4)	0 (4)	0 (4)	0 (4)	0.5 (4)	5 (3)	0 (5)
	Advanced training methods	0 (1)	0 (2)	0 (3)	0.5 (3)	3 (2)	0.5 (5)	4 (4)	4 (3)
	Equipment	0 (1)	0 (2)	0 (1)	0 (2)	0 (4)	0 (4)	0 (4)	0 (5)
	Recovery strategies	0 (2)	0 (2)	0 (2)	0 (2)	3.5 (4)	2.5 (4)	4 (3)	4 (5)
	Nutrition	0 (1)	0 (2)	1.5 (3)	0 (2)	3.5 (4)	2 (4)	4 (4)	4 (4)
Summary Rating	**< 3**	**n = 7**	**n = 7**	**n = 7**	**n = 7**	**n = 3**	**n = 7**	**n = 2**	**n = 4**
	**3**	**n = 0**	**n = 0**	**n = 0**	**n = 0**	**n = 1**	**n = 0**	**n = 1**	**n = 0**
	**> 3**	**n = 0**	**n = 0**	**n = 0**	**n = 0**	**n = 3**	**n = 0**	**n = 4**	**n = 3**

S-TABLE 9. Continue.

		Significance testing (different prompt, different AI) – random							
	**Relevant aspects when designing a training plan**	**GGL1 vs GPT-4D1**	**GGL1 vs GPT-4D2**	**GGL2 vs GPT-4D1**	**GGL2 vs GPT-4D2**	**GPT-4L1 vs GGD1**	**CL1 vs GD2**	**CL2 vs GD1**	**C2 vs GD2**
General Aspects	Overall training plan	**0.013**	0.391	**0.00**	**0.008**	1.000	1.000	1.000	1.000
	Health screening	1.000	1.000	1.000	1.000	1.000	1.000	1.000	1.000
	Defined goal	**0.018**	0.066	0.113	0.348	0.391	1.000	1.000	1.000
	Overall testing procedure	1.000	1.000	1.000	1.000	1.000	1.000	1.000	1.000
	Testing procedure regarding initial performance status	1.000	1.000	1.000	1.000	1.000	1.000	1.000	1.000
	Testing procedure regarding assessment of individual training variables	1.000	1.000	1.000	1.000	1.000	1.000	1.000	1.000
	Testing procedure regarding assessment of training effects	1.000	1.000	1.000	1.000	1.000	1.000	1.000	1.000
	Overall monitoring procedure	1.000	1.000	1.000	1.000	1.000	1.000	1.000	1.000
Summary Rating	< 3								
	3								
	> 3								
Training principles	Principle of specificity	**0.001**	0.309	**0.000**	0.129	1.000	1.000	1.000	1.000
	Principle of progressive overload	1.000	0.099	1.000	1.000	1.000	1.000	1.000	1.000
	Principle of variation	0.940	**0.050**	0.076	0.002	1.000	1.000	1.000	1.000
	Principle of recovery	0.847	0.129	1.000	0.391	1.000	1.000	1.000	1.000
Summary Rating	< 3								
	3								
	> 3								
Basic strength training aspects	Exercise selection	**0.024**	0.215	**0.001**	**0.003**	1.000	1.000	1.000	1.000
	Exercise order	1.000	1.000	**0.002**	**0.001**	0.550	1.000	0.274	1.000
	Weekly training frequency per muscle	0.348	0.309	0.243	0.215	1.000	1.000	1.000	1.000
	Training intensity per exercise	0.491	1.000	0.391	1.000	1.000	1.000	1.000	1.000
	Repetition range per exercise	0.762	1.000	**0.028**	1.000	1.000	1.000	0.491	1.000
	Overall training volume	1.000	1.000	**0.009**	**0.004**	1.000	1.000	1.000	1.000
	Number of sets per muscle per week	1.000	1.000	1.000	**0.008**	1.000	1.000	1.000	1.000
	Rest periods	1.000	1.000	**0.000**	**0.002**	1.000	1.000	1.000	1.000
	Exercise technique	1.000	1.000	1.000	1.000	1.000	1.000	1.000	1.000
Summary Rating	< 3								
	3								
	> 3								
Advanced training aspects	Advanced exercise methods	0.439	1.000	0.309	1.000	**0.015**	1.000	0.274	1.000
	Time under tension	1.000	1.000	1.000	1.000	0.348	1.000	0.550	1.000
	Set end point	**0.043**	1.000	**0.015**	1.000	1.000	1.000	1.000	1.000
	Advanced training methods	**0.000**	**0.000**	**0.000**	**0.000**	**0.028**	1.000	0.391	1.000
	Equipment	1.000	1.000	1.000	1.000	1.000	1.000	1.000	1.000
	Recovery strategies	**0.000**	**0.001**	**0.000**	**0.001**	0.087	1.000	**0.028**	1.000
	Nutrition	**0.000**	**0.000**	**0.000**	**0.000**	0.762	1.000	**0.028**	0.189
Summary Rating	< 3								
	3								
	> 3								

GGL1 = Google Gemini, little information, first try; GGL2 = Google Gemini, little information, second try; GPT-4L1 = GPT-4, little information, first try; GPT-4L2 = GPT-4, little information, second try; GGD1 = Google Gemini, detailed information, first try; GGD2 = Gemini, detailed information, second try; GPT-4D1 = GPT-4, detailed information, first try; GPT-4D2 = GPT-4, detailed information, second try.

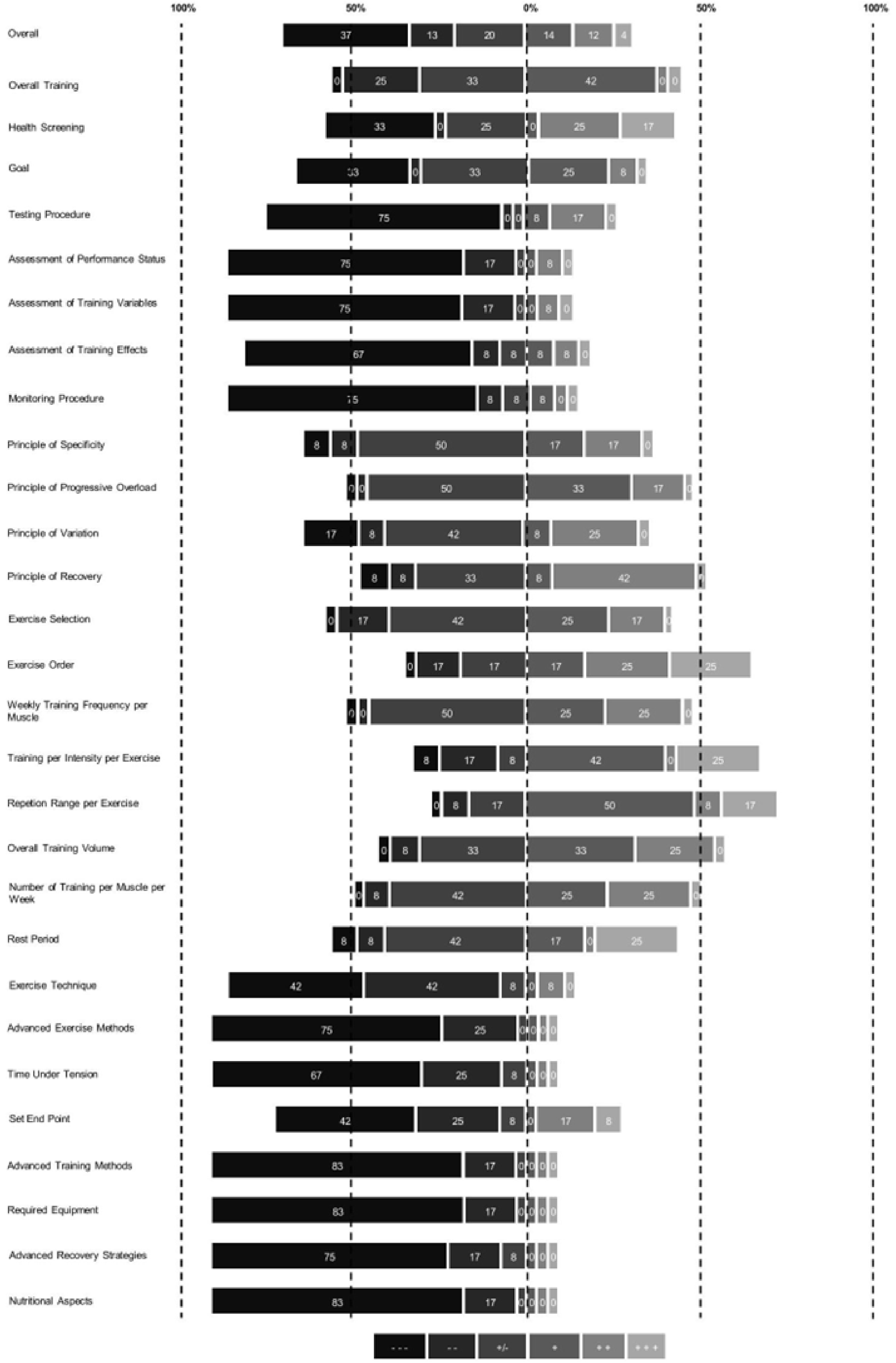

S-FIGURE 1. Likert Scale Chart of Google Gemini with little information input (first attempt)

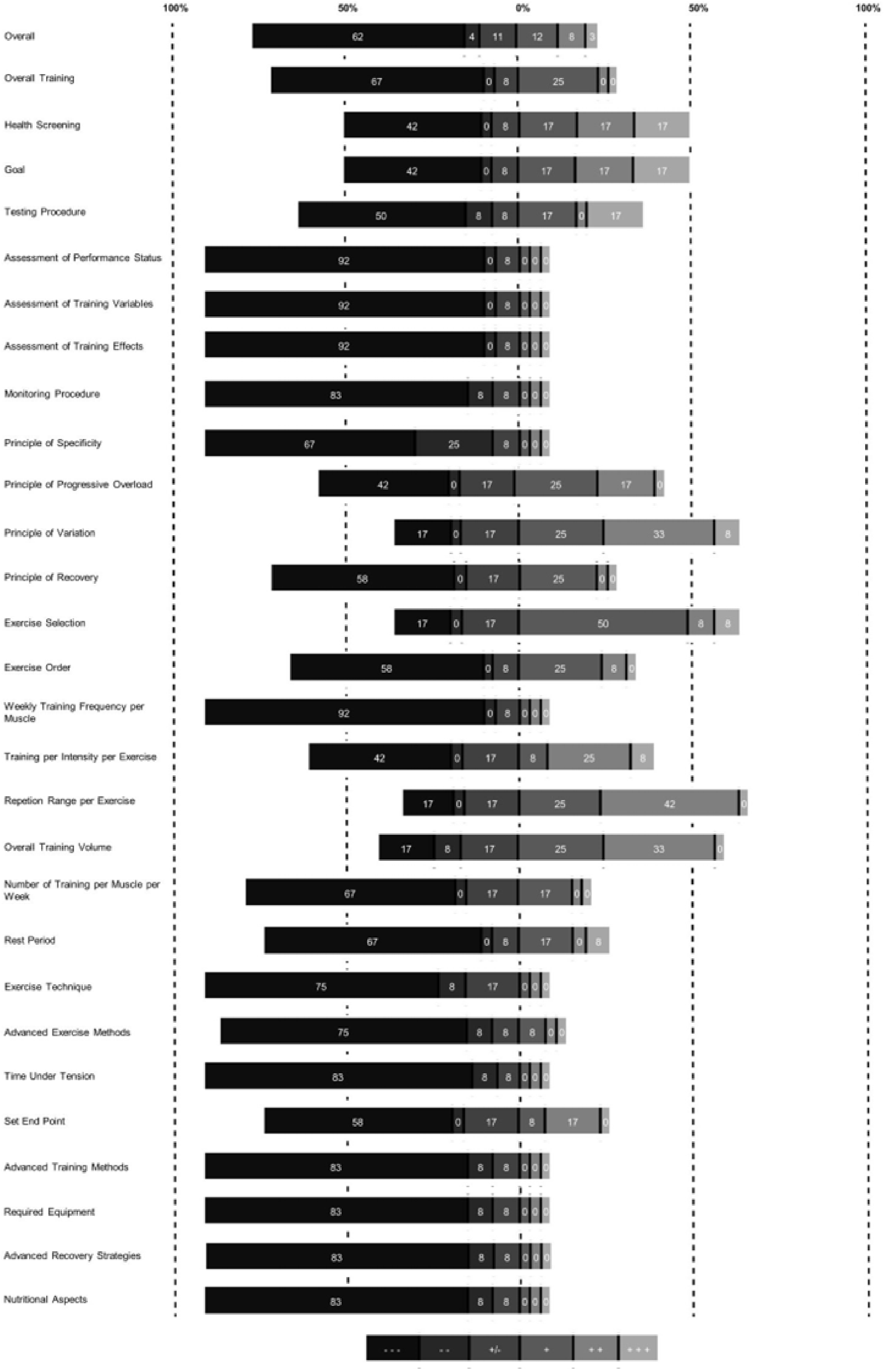

S-FIGURE 2. Likert Scale Chart of Google Gemini with little information input (second attempt)

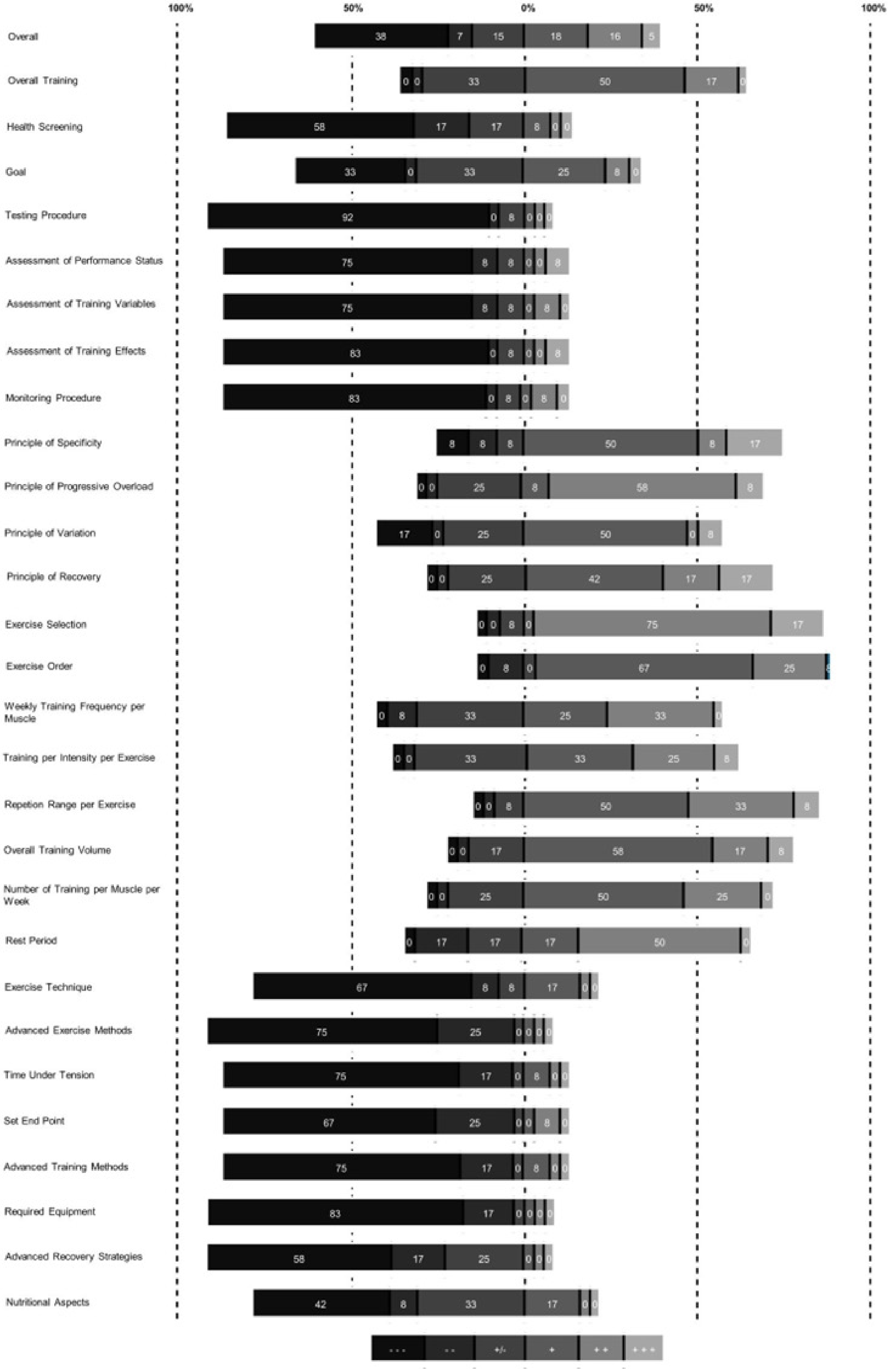

S-FIGURE 3. Likert Scale Chart of Microsoft Copilot with little information input (first attempt)

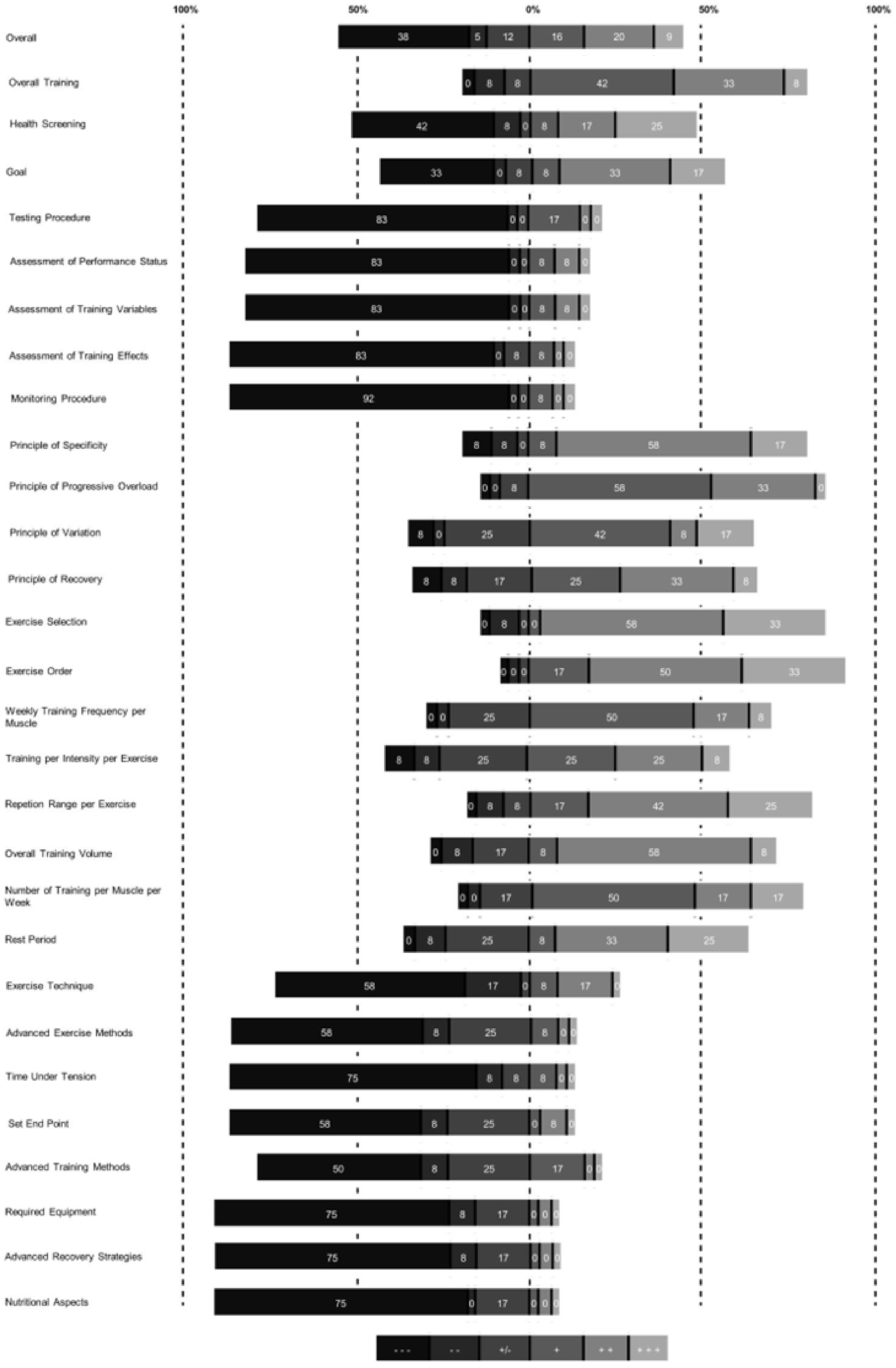

S-FIGURE 4. Likert Scale Chart of Microsoft Copilot with little information input (second attempt)

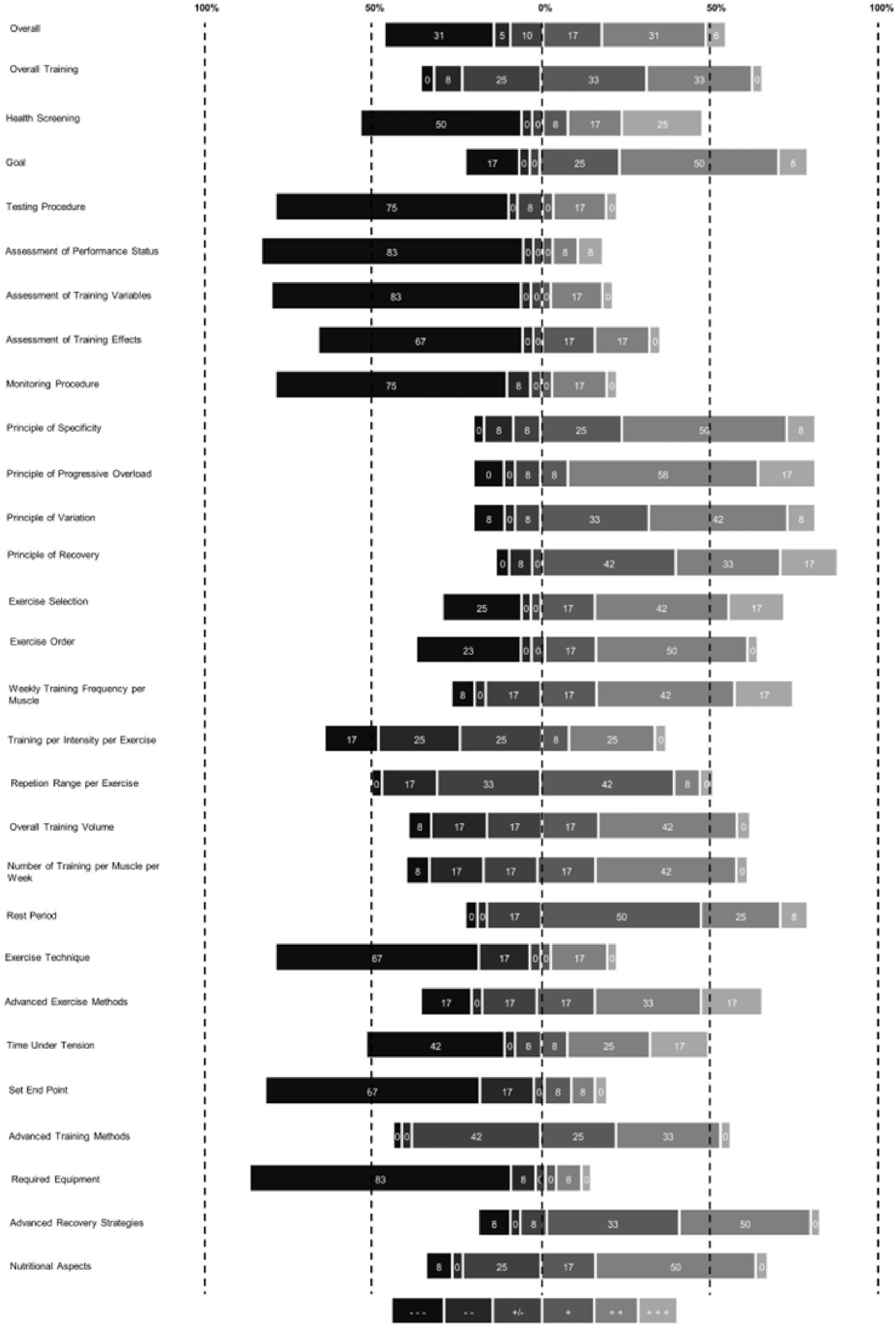

S-FIGURE 5. Likert Scale Chart of Google Gemini with detailed information input (first attempt)

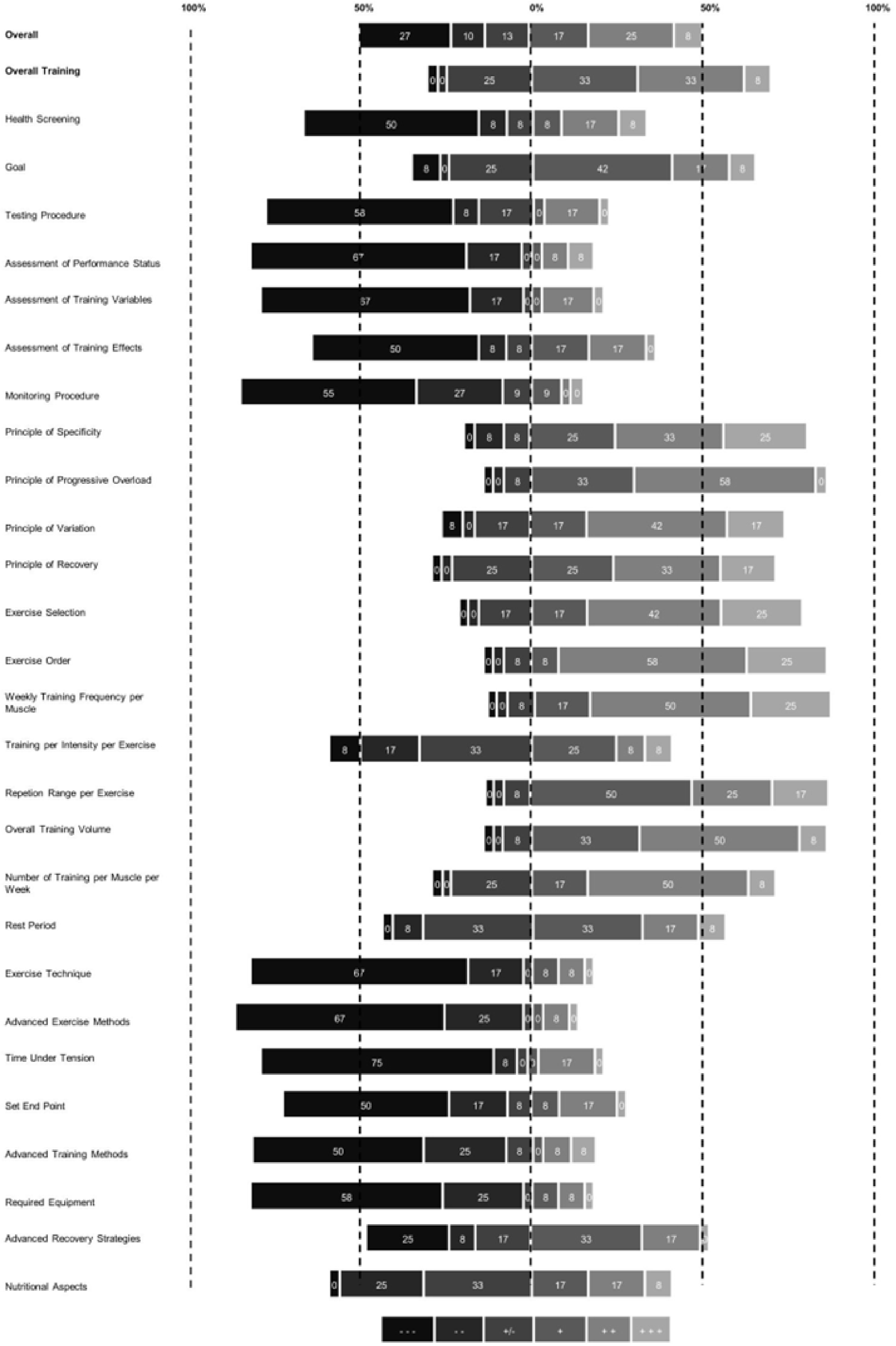

S-FIGURE 6. Likert Scale Chart of Google Gemini with detailed information input (second attempt)

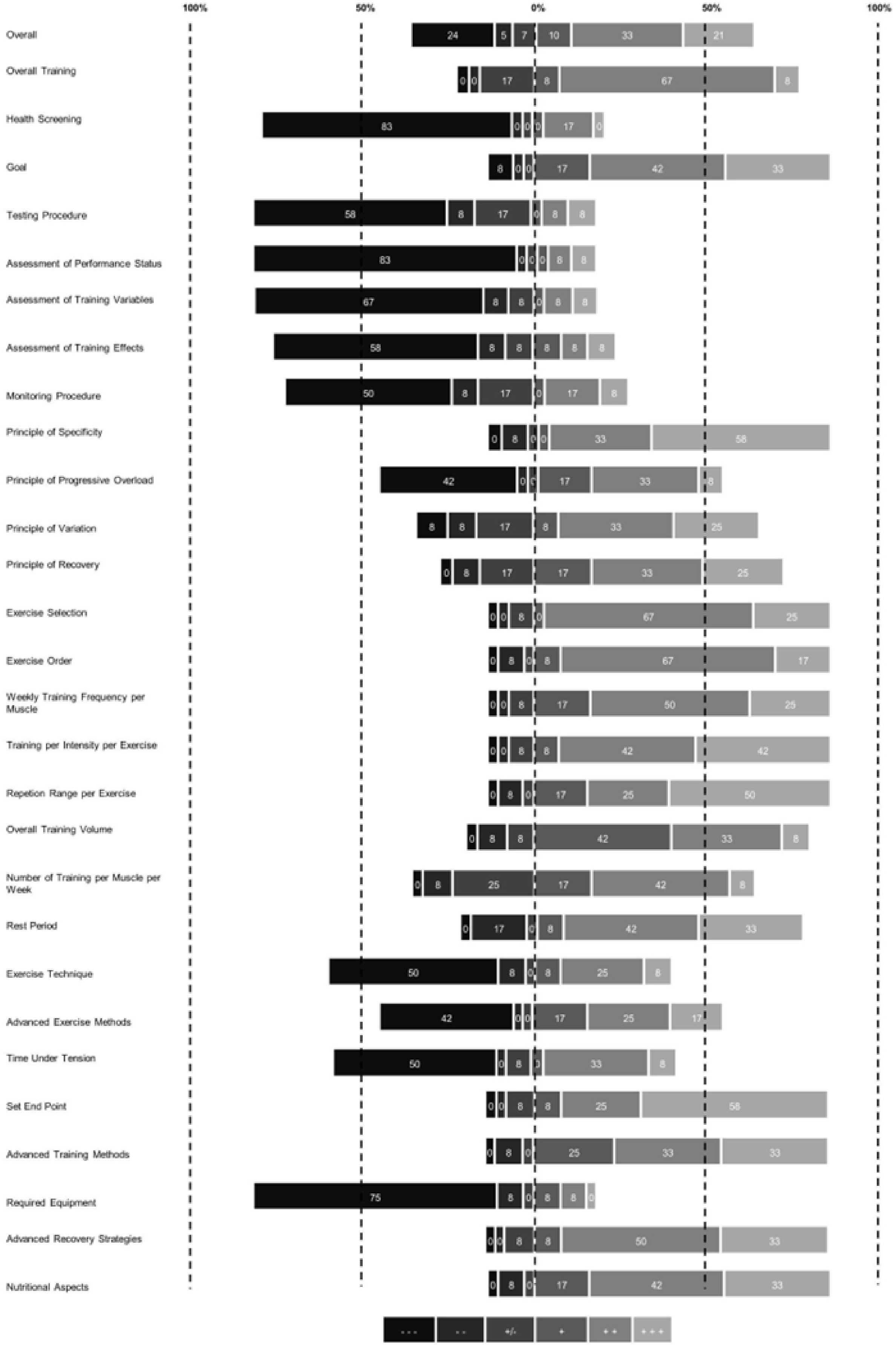

S-FIGURE 7. Likert Scale Chart of Microsoft Copilot with detailed information input (first attempt)

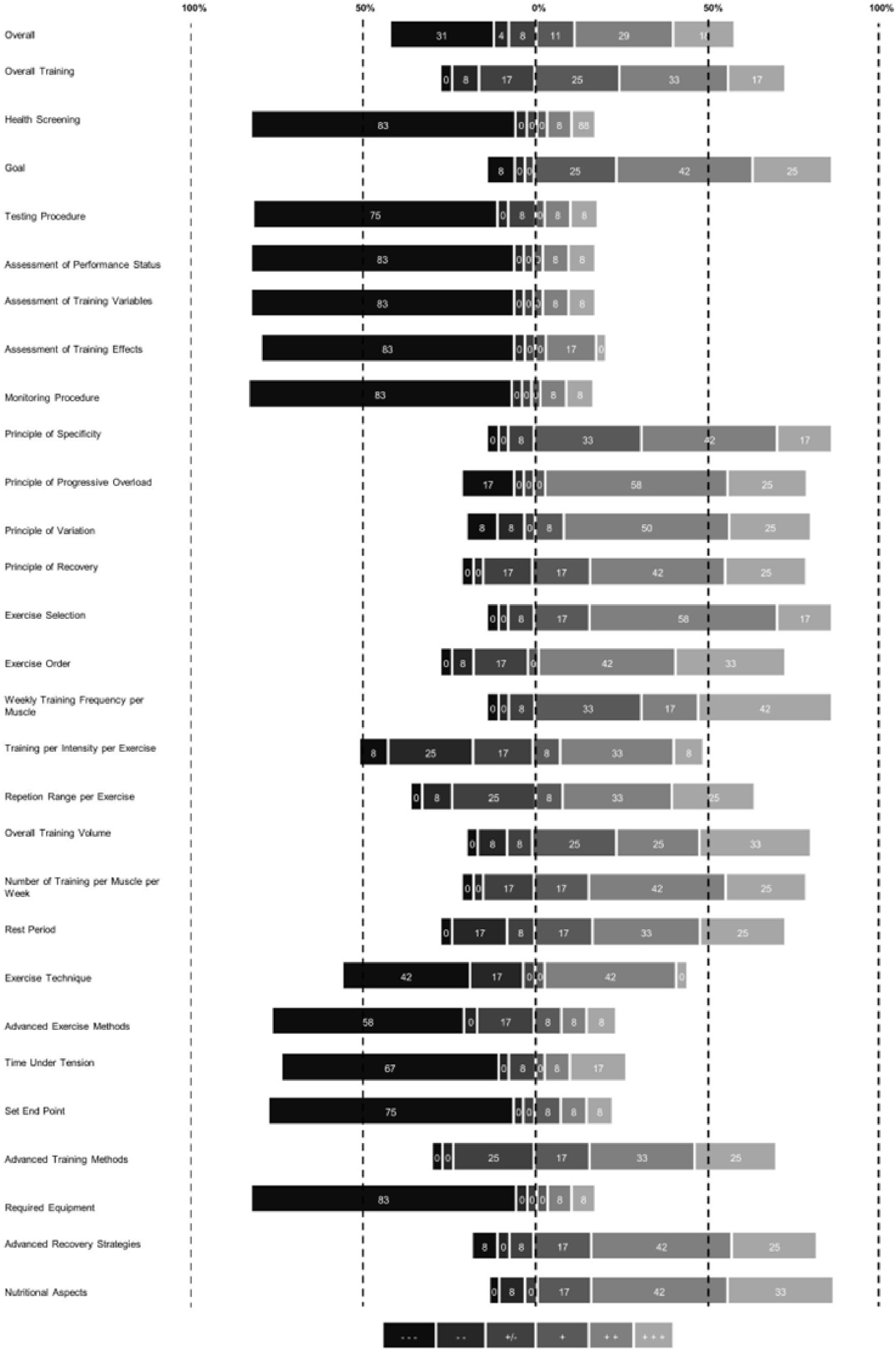

S-FIGURE 8. Likert Scale Chart of Microsoft Copilot with detailed information input (second attempt)
